# Exploring prognostic factors in breast cancer: development and selection of optimal machine learning models

**DOI:** 10.3389/fonc.2026.1830769

**Published:** 2026-07-28

**Authors:** Meiying Shen, Yulei Wang, Zongyuan Wu, Lifei Chen, Shaofeng Wu, Huawen Pan, Bo Xu

**Affiliations:** 1 Department of Breast Surgery, Maoming People’s Hospital, Maoming, China; 2 Department of Breast Surgery, Guangdong Provincial People’s Hospital, Guangzhou, China; 3 Department of Ultrasound, Maoming People’s Hospital, Maoming, China; 4 Department of Spine Surgery, Maoming People’s Hospital, Maoming, China; 5 Department of Breast and Thyroid Surgery, Guangzhou First People’s Hospital, Guangzhou, China

**Keywords:** breast cancer, prognostic factors, machine learning, random forest, XGBoost, SHAP interpretation, survival analysis

## Abstract

**Objective:**

To investigate prognostic factors for breast cancer recurrence and metastasis, and to systematically compare multiple machine learning models to develop an optimal predictive tool.

**Methods:**

We retrospectively analyzed data from 1,056 breast cancer patients diagnosed between January 2012 and June 2021 at a single center. Patients were randomly divided into a training (n=740) and a validation (n=316) set. Univariate and multivariate Cox proportional hazards regression analyses were performed to identify independent prognostic factors. Seven machine learning algorithms (Cox regression, LASSO, Elastic-Net, Decision Tree, Random Forest, XGBoost, and GBM) were employed. All models were implemented using survival-specific adaptations. A rigorous 5-fold cross-validation framework was used for model training and hyperparameter tuning. Model performance was evaluated using time-dependent Area Under the Curve (AUC), Brier scores, calibration curves, calibration-in-the-large, calibration slopes, and Decision Curve Analysis (DCA). SHAP values were employed for model interpretation.

**Results:**

Multivariate Cox regression revealed that tumor size (cm) (HR = 1.025, 95%CI: 1.013-1.037), lymph node dissection (HR = 0.278, 95%CI: 0.199-0.389), ER% (HR = 1.006, 95%CI: 1.003-1.009), PR% (HR = 1.005, 95%CI: 1.002-1.008), Ki-67% (HR = 1.012, 95%CI: 1.007-1.016), and HER2 status (HR = 1.195, 95%CI: 1.098-1.301) were independently associated with disease-free survival. Random Forest and XGBoost demonstrated superior and stable predictive performance, with Random Forest achieving time-dependent AUCs of 0.851 (95%CI: 0.802-0.900, 1-year), 0.763 (95%CI: 0.702-0.824, 3-year), and 0.826 (95%CI: 0.766-0.886, 5-year) in the validation set. The Brier scores for Random Forest were consistently low, and calibration metrics confirmed excellent calibration. DCA indicated a positive net benefit across a wide range of threshold probabilities.

**Conclusion:**

This single-center study identifies key prognostic factors for breast cancer and demonstrates that ensemble machine learning models, particularly Random Forest, offer superior predictive power. The integration of SHAP interpretation provides a methodological framework for potential clinical application. However, all predictive models require external, multi-center validation before clinical consideration. These findings provide a promising methodological basis, but caution is warranted against overinterpretation until independent verification is complete.

## Introduction

Breast cancer is the most commonly diagnosed malignancy and the leading cause of cancer-related death among women worldwide (1, 2). Despite significant advances in early detection and treatment modalities, considerable heterogeneity exists in patient outcomes, with recurrence and metastasis remaining major challenges in clinical management (3, 4). Accurate prognostic assessment is essential for individualized treatment planning and improving patient survival.

Traditional prognostic factors for breast cancer include tumor size, lymph node status, histological grade, molecular subtypes (ER, PR, HER2, Ki-67), and lymphovascular invasion (5–7). While these factors provide valuable prognostic information, their predictive accuracy is limited when considered in isolation or through conventional statistical methods (8). The complex interplay between clinical, pathological, and immunological factors necessitates more sophisticated analytical approaches.

In recent years, inflammation-based prognostic indicators have garnered increasing attention in oncology research (9, 10). Biomarkers such as Neutrophil-to-lymphocyte ratio (NLR), Platelet-to-lymphocyte ratio (PLR), and Systemic Immune-Inflammation Index (SII) reflect the tumor microenvironment’s inflammatory status and have been identified as independent prognostic factors in various malignancies, including breast cancer (11, 12). These indicators capture the complex interactions between tumor cells and the host immune system, potentially offering additional prognostic value beyond traditional clinicopathological parameters (13).

Machine learning algorithms have emerged as powerful tools for integrating multidimensional data and capturing complex non-linear relationships in medical research (14, 15). Unlike conventional regression models that assume linear relationships and limited interactions, machine learning methods can automatically identify patterns, handle high-dimensional data, and improve prediction accuracy (16, 17). Algorithms such as Random Forest, XGBoost, and Support Vector Machines have shown promise in cancer prognosis prediction, often outperforming traditional statistical methods (18, 19).

However, several challenges remain in applying machine learning to breast cancer prognosis. First, most existing models focus on a limited set of predictors or single algorithmic approaches (20, 21). Second, model interpretability remains a significant barrier to clinical adoption, as clinicians require understanding of how predictions are generated (22). Third, external validation and comparison of multiple algorithms in the same cohort are relatively scarce (23).

To address these gaps, this study aims to: (1) comprehensively investigate prognostic factors for breast cancer recurrence and metastasis, including traditional clinicopathological features and inflammatory markers; (2) systematically compare seven machine learning algorithms for prognostic prediction; (3) develop and internally validate optimal prediction models for 1-year, 3-year, and 5-year outcomes; and (4) enhance model interpretability using SHAP (SHapley Additive exPlanations) values to facilitate clinical translation. By integrating multi-modal data and advanced analytical methods, we propose a promising methodological framework that may support future individualized risk stratification research in breast cancer, pending external validation.

## Methods

### Study population

#### Patient selection

This retrospective study consecutively enrolled 1,056 patients diagnosed with breast cancer at the Department of Breast Surgery, Maoming People’s Hospital between January 2012 and June 2021. The study protocol was approved by the Clinical Database Committee of Maoming People’s Hospital, and a data confidentiality agreement was signed. All personal identifying information was removed prior to analysis. The study was conducted in accordance with the Declaration of Helsinki.

#### Inclusion criteria

1. Histopathologically confirmed unilateral primary breast cancer without distant metastasis.
2. Received treatment at our institution.
3. Underwent breast surgery.
4. Underwent breast tumor color Doppler ultrasound within 2 weeks before surgery.
5. Completed peripheral blood laboratory examination within 2 days before surgery.
6. Complete clinical data without significant missing values.

#### Exclusion criteria

1. No surgical pathology results.
2. Poor quality of grayscale ultrasound, color Doppler, or elastography images.
3. Diagnosis of other malignancies within the past 3 months or inflammatory diseases within the past 2 weeks.
4. Follow-up duration less than 6 months or lost to follow-up.
5. Concurrent other malignant tumors.
6. Comorbid immune or hematological disorders.
7. Recent infectious or traumatic conditions.
8. Pregnant or lactating patients.
9. Incomplete clinical or pathological data.

### Study procedures

#### Ultrasound examination

All breast and axillary ultrasound examinations were performed by a single experienced ultrasound specialist with over 15 years of experience in breast ultrasound diagnosis. Ultrasound examinations were conducted using a HITACHI EUB-8500 system (Hitachi Medical Corporation, Tokyo, Japan) with a probe frequency of 7.5–13 MHz.

#### Clinical and pathological data collection

Data were extracted from inpatient electronic medical records and outpatient visit systems, including:

- Patient characteristics: Age, menopausal status (premenopausal, perimenopausal, postmenopausal), family history of breast cancer.
- TNM staging: Based on AJCC 8th edition, including T stage and N stage information.
- Axillary lymph node (ALN) metastasis classification: pN0 (no metastasis), pN1 (1–3 metastases), pN2 (4–9 metastases), pN3 (≥10 metastases).
- Surgical procedures: Radical mastectomy or breast-conserving surgery with sentinel lymph node biopsy and/or axillary lymph node dissection.
- Pathological features: Pathological subtype, molecular subtype, tumor size, multifocality, lymphovascular invasion (LVI) status, histological grade, and ultrasound features (spiculation, aspect ratio, blood flow).
- Laboratory indicators: Preoperative (within 3 days) platelet count, lymphocyte count, monocyte count, neutrophil count, and tumor markers.
- Inflammatory indices: NLR = Neutrophil count/Lymphocyte count; PLR = Platelet count/Lymphocyte count; LMR = Lymphocyte count/Monocyte count; SII = Platelet count × Neutrophil count/Lymphocyte count.
- Tumor markers: CA199, CEA, CA725, CA724, CA125 (positive if any marker elevated).

### Pathological assessment

All specimens were diagnosed by pathologists with over 5 years of experience. Pathological tumor size was measured on gross specimens. Immunohistochemical staining was performed to assess estrogen receptor (ER), progesterone receptor (PR), human epidermal growth factor receptor 2 (HER2), and Ki-67 expression. ER and PR negativity was defined as <1% nuclear staining in tumor cells. HER2 negativity was defined as IHC score 0 or 1+, or negative by fluorescence *in situ* hybridization (FISH). Breast cancer molecular subtypes were classified as Luminal A, Luminal B, HER2-enriched, and triple-negative breast cancer (TNBC). TNBC was defined as negative expression of ER, PR, and HER2. ER and PR positivity was defined as expression >1%. Ki-67 expression was dichotomized at the 14% cutoff. Androgen receptor (AR) positivity was defined as expression >1%.

### Follow-up

Postoperative treatment regimens were determined based on surgical approach, molecular subtype, and axillary lymph node status, including: radiotherapy + chemotherapy + endocrine therapy, chemotherapy + endocrine therapy, chemotherapy + targeted therapy + endocrine therapy, and traditional Chinese medicine.

Follow-up frequency and examinations followed the Chinese Anti-Cancer Association guidelines: every 3 months during the first 2 years post-surgery, every 6 months during years 3-5, and annually after 5 years. Monitoring included laboratory tests, breast and axillary ultrasound and/or mammography, abdominal and gynecological ultrasound, CT or MRI, bone scan, and whole-body PET-CT as indicated for distant metastasis surveillance.

The final follow-up date was November 20, 2024. Recurrence was classified as local recurrence (confined to ipsilateral breast, chest wall, and/or axillary, infraclavicular, or supraclavicular lymph nodes), contralateral breast, or distant metastasis. The endpoint event was defined as recurrence or metastasis during postoperative follow-up. Disease-free survival (DFS) was defined as the time from initial surgical diagnosis to the first diagnosis of any site recurrence or metastasis, death from any cause, or last follow-up date, whichever occurred first.

### Statistical analysis

Statistical analyses were performed using R software (version 4.2.1) and IBM SPSS Statistics 26.0.

### Data splitting and preprocessing

The dataset was randomly divided into a training set (70%, n=740) and a validation set (30%, n=316). Stratified sampling based on the event indicator was used to maintain identical event proportions in both partitions, with the seed fixed at 2023 via the createDataPartition function of the caret package. All preprocessing steps, including missing data imputation, feature selection, and hyperparameter tuning, were performed exclusively within the training set and subsequently applied to the validation set. Missing values were handled using multiple imputation with the mice package, where the imputation model was estimated on the training set only. Within cross-validation loops, a simplified single imputation derived from the respective training fold was employed to maintain computational efficiency; the stability of imputed values across folds was confirmed. After imputation, continuous variables were standardized (z-score normalization) based on the training set mean and standard deviation.

Normality of continuous variables was assessed using the Shapiro-Wilk test. Normally distributed data were expressed as mean ± standard deviation and compared using t-tests. Non-normally distributed data were expressed as median (interquartile range) and compared using Wilcoxon rank-sum tests. Categorical variables were expressed as frequencies (percentages) and compared using chi-square tests or Fisher’s exact tests.

### Predictor selection

To maintain methodological consistency with the time-to-event outcome (DFS), we employed univariate and multivariate Cox proportional hazards regression analyses. Variables with P < 0.05 in the multivariate Cox analysis were identified as independent prognostic factors and subsequently used as input features for all seven machine learning models. Recognizing the inherent limitations of significance-based variable selection, we also performed a supplementary analysis using Cox LASSO with 10-fold cross-validation. LASSO selected the same six core variables with the addition of SII, but model performance did not improve appreciably (increase in C-index <0.01), and the ranking of variable importance remained similar. Therefore, the six clinically established and parsimonious variables were retained to ensure interpretability and comparability across models. The potential instability of P-value-based selection is acknowledged and addressed further in the Discussion.

### Model development and survival-specific implementations

Seven machine learning algorithms were compared. Crucially, to ensure valid handling of censored time-to-event data, all models employed survival-adapted implementations, never reducing the endpoint to binary classification at fixed time points. The specific implementations were as follows:

- Cox proportional hazards model: Standard Cox regression as implemented in the survival R package.
- LASSO (Least Absolute Shrinkage and Selection Operator) Regression: Cox family in glmnet (via family = “cox”), which directly optimizes the penalized partial likelihood.
- Elastic-Net: Also implemented via glmnet with family = “cox”, combining L1 and L2 penalties.
- Decision Tree: Survival trees were constructed using conditional inference trees (partykit::ctree), with splitting based on the log-rank test statistic, inherently accommodating censored data.
- Random Forest: Random Survival Forest as implemented in the randomForestSRC package. Splits were guided by the log-rank statistic, and predictions were provided as ensemble survival functions, from which time-dependent probabilities can be derived.
- XGBoost (eXtreme Gradient Boosting): Trained with the objective function objective = “survival:cox” in the xgboost package, which optimizes the Cox partial likelihood. The model outputs the linear predictor and cumulative hazard, fully incorporating censored observations.
- GBM (Gradient Boosting Machines): Implemented with distribution = “coxph” in the gbm package, likewise optimizing the Cox partial likelihood.

All models were supplied with the identical set of six predictor variables identified from the multivariate Cox regression.

### Hyperparameter tuning and internal validation

An inner 5-fold cross-validation repeated 10 times (5×10 repeated CV) was performed exclusively on the training set to tune hyperparameters. The combination that maximized the time-dependent concordance index (C-index) was selected. After optimal hyperparameters were identified, the final model was refit on the complete training set and then evaluated once on the untouched validation set to yield unbiased performance estimates. All resampling procedures used a fixed random seed to guarantee full reproducibility.

### Model evaluation

Model performance was assessed using the following metrics:

- Time-dependent Receiver Operating Characteristic (ROC) curves and Area Under the Curve (AUC): To assess discrimination at 1-year, 3-year, and 5-year time points. 95% confidence intervals for AUCs were computed via bootstrapping.
- Concordance index (C-index): To evaluate the overall discriminative ability of survival models.
- Brier score: To assess the accuracy of probabilistic predictions (calibration), with lower scores indicating better calibration.
- Calibration curves: To visually evaluate prediction accuracy against observed outcomes at 1, 3, and 5 years. Additionally, calibration-in-the-large and calibration slope were calculated to provide numerical measures of calibration.
- Decision Curve Analysis (DCA): To quantify net benefit across decision thresholds and assess potential clinical utility.

### Model interpretation

Model interpretability was enhanced using SHAP (SHapley Additive exPlanations) values to identify the most influential variables and their direction of impact on model predictions. For survival models, SHAP values were derived from the predicted risk scores.

## Results

### General characteristics

Comparison between training (n=740) and validation (n=316) sets revealed significant differences in 3 of 28 variables (p < 0.05), including PR (p = 0.009) and pathological tumor diameter (p < 0.001). No significant differences were observed for other variables such as diabetes, hyperlipidemia, biopsy status, or histological grade (p > 0.05) (**Table 1**).

**Table 1:** Comparison of clinical characteristics between training and validation sets.

Characteristic	Train (n=740)	Validation (n=316)	Total (n=1056)	P value
Age, median (IQR)	53 (46,60)	52.5 (46,59)	53 (46,59)	0.353
Diabetes				0.22
- No	707 (95.5)	307 (97.2)	1014 (96)	
- Yes	33 (4.5)	9 (2.8)	42 (4)	
Hyperlipidemia				0.313
- No	715 (96.6)	309 (97.8)	1024 (97)	
- Yes	25 (3.4)	7 (2.2)	32 (3)	
Hypertension				0.21
- No	679 (91.8)	297 (94)	976 (92.4)	
- Yes	61 (8.2)	19 (6)	80 (7.6)	
BMI, median (IQR)	23.1 (21.3,25.3)	23.4 (21.7,25.4)	23.2 (21.5,25.3)	0.084
Prior tumor history				0.672
- No	678 (91.6)	287 (90.8)	965 (91.4)	
- Yes	62 (8.4)	29 (9.2)	91 (8.6)	
BI-RADS, median (IQR)	4 (4,4)	4 (4,4)	4 (4,4)	0.746
Size (cm), median (IQR)	2.6 (1.8,3.9)	2.5 (1.6,4)	2.5 (1.7,4)	0.463
Laterality				0.375
- Left	326 (44.1)	154 (48.7)	480 (45.5)	
- Right	354 (47.8)	139 (44)	493 (46.7)	
- Bilateral	60 (8.1)	23 (7.3)	83 (7.9)	
Aspect ratio (>1 = 1/<1 = 0)				0.725
- 0	583 (78.8)	252 (79.7)	835 (79.1)	
- 1	157 (21.2)	64 (20.3)	221 (20.9)	
Spiculation				0.631
- No	244 (33)	109 (34.5)	353 (33.4)	
- Yes	496 (67)	207 (65.5)	703 (66.6)	
Blood flow				0.67
- No	289 (39.1)	119 (37.7)	408 (38.6)	
- Yes	451 (60.9)	197 (62.3)	648 (61.4)	
Axillary lymph nodes				0.351
- No	460 (62.2)	206 (65.2)	666 (63.1)	
- Yes	280 (37.8)	110 (34.8)	390 (36.9)	
Tumor location				0.56
- 0	5 (0.7)	2 (0.6)	7 (0.7)	
- Upper inner	154 (20.8)	55 (17.4)	209 (19.8)	
- Upper outer	434 (58.6)	203 (64.2)	637 (60.3)	
- Lower inner	49 (6.6)	19 (6)	68 (6.4)	
- Lower outer	98 (13.2)	37 (11.7)	135 (12.8)	
Biopsy (time)				0.843
- No	403 (54.5)	170 (53.8)	573 (54.3)	
- Yes	337 (45.5)	146 (46.2)	483 (45.7)	
Distant metastasis				0.999
- No	644 (87)	275 (87)	919 (87)	
- Yes	96 (13)	41 (13)	137 (13)	
Breast-conserving surgery				0.288
- No	482 (65.1)	195 (61.7)	677 (64.1)	
- Yes	258 (34.9)	121 (38.3)	379 (35.9)	
Lymph node dissection				0.638
- No	629 (85)	265 (83.9)	894 (84.7)	
- Yes	111 (15)	51 (16.1)	162 (15.3)	
Pathological tumor diameter (cm), median (IQR)	1.9 (1.3,3)	1.6 (0.9,2.5)	1.8 (1.2,3)	<0.001
Histological grade				0.431
- 0	36 (4.9)	18 (5.7)	54 (5.1)	
- G1	107 (14.5)	50 (15.8)	157 (14.9)	
- G2	349 (47.2)	161 (50.9)	510 (48.3)	
- G3	217 (29.3)	75 (23.7)	292 (27.7)	
- G4	31 (4.2)	12 (3.8)	43 (4.1)	
Lymph node status, median (IQR)	0 (0,0.1)	0 (0,0.2)	0 (0,0.1)	0.909
Lymphovascular invasion				0.1
- No	563 (76.1)	255 (80.7)	818 (77.5)	
- Yes	177 (23.9)	61 (19.3)	238 (22.5)	
ER (%), median (IQR)	80 (5,90)	72.5 (0,90)	80 (4.5,90)	0.404
PR (%), median (IQR)	37.5 (0,80)	22.5 (0,70)	30 (0,80)	0.009
Ki-67 (%), median (IQR)	35 (20,60)	31.5 (20,60)	35 (20,60)	0.695
HER2				0.618
- 0	280 (37.8)	112 (35.4)	392 (37.1)	
- 1	139 (18.8)	68 (21.5)	207 (19.6)	
- 2	128 (17.3)	49 (15.5)	177 (16.8)	
- 3	193 (26.1)	87 (27.5)	280 (26.5)	
Adjuvant therapy				0.696
- No	101 (13.6)	46 (14.6)	147 (13.9)	
- Yes	639 (86.4)	270 (85.4)	909 (86.1)	
Palliative therapy				0.535
- No	703 (95)	303 (95.9)	1006 (95.3)	
- Yes	37 (5)	13 (4.1)	50 (4.7)	
Chemotherapy				0.957
- No	219 (29.6)	93 (29.4)	312 (29.5)	
- Yes	521 (70.4)	223 (70.6)	744 (70.5)	
Endocrine therapy				0.438
- No	340 (45.9)	137 (43.4)	477 (45.2)	
- Yes	400 (54.1)	179 (56.6)	579 (54.8)	
Targeted therapy				0.592
- No	525 (70.9)	219 (69.3)	744 (70.5)	
- Yes	215 (29.1)	97 (30.7)	312 (29.5)	
Immunotherapy				0.811
- No	696 (94.1)	296 (93.7)	992 (93.9)	
- Yes	44 (5.9)	20 (6.3)	64 (6.1)	
Pathological type				0.169
- Invasive ductal carcinoma	668 (90.3)	275 (87)	943 (89.3)	
- Invasive lobular carcinoma	9 (1.2)	9 (2.8)	18 (1.7)	
- Carcinoma in situ	28 (3.8)	17 (5.4)	45 (4.3)	
- Other	35 (4.7)	15 (4.7)	50 (4.7)	
Outcome				0.096
- Event-free	329 (44.5)	123 (38.9)	452 (42.8)	
- Event	411 (55.5)	193 (61.1)	604 (57.2)	

### Baseline characteristics by outcome

Comparison between event-free (n=452) and event (n=604) groups revealed significant differences in multiple variables. Prior tumor history was significantly higher in the event group (6.4% vs. 10.3%, P = 0.027). BI-RADS category showed significant differences between groups (P = 0.029). Ultrasound-measured tumor size was significantly smaller in the event group (median 2.3 cm vs. 2.8 cm, P = 0.007). This counterintuitive finding is likely attributable to differences in surgical and pathological assessment; the event group had a higher rate of breast-conserving surgery, and gross pathological measurement may systematically differ from preoperative imaging, particularly after neoadjuvant therapy or in multifocal disease which was subsequently pathologically confirmed. Pathological tumor diameter was also significantly smaller in the event group (median 1.5 cm vs. 2.5 cm, P<0.001). Additionally, significant differences were observed in lymphovascular invasion, HER2 status, adjuvant therapy, chemotherapy, and immunotherapy (P<0.05) (**Table 2**).

**Table 2:** Baseline characteristics by outcome status.

Characteristic	Event-free (n=452)	Event (n=604)	Total (n=1056)	P value
Age, median (IQR)	52 (46,59)	53 (46,59.2)	53 (46,59)	0.374
Diabetes				0.529
- No	436 (96.5)	578 (95.7)	1014 (96)	
- Yes	16 (3.5)	26 (4.3)	42 (4)	
Hyperlipidemia				0.088
- No	443 (98)	581 (96.2)	1024 (97)	
- Yes	9 (2)	23 (3.8)	32 (3)	
Hypertension				0.377
- No	414 (91.6)	562 (93)	976 (92.4)	
- Yes	38 (8.4)	42 (7)	42 (7.6)	
BMI, median (IQR)	23.4 (21,25)	23.1 (21.6,25.4)	23.2 (21.5,25.3)	0.081
Prior tumor history				0.027
- No	423 (93.6)	542 (89.7)	965 (91.4)	
- Yes	29 (6.4)	62 (10.3)	91 (8.6)	
BI-RADS, median (IQR)	4 (4,4)	4 (4,4)	4 (4,4)	0.029
Size (cm), median (IQR)	2.8 (2,3.8)	2.3 (1.5,4)	2.5 (1.7,4)	0.007
Laterality				0.663
- Left	207 (45.8)	273 (45.2)	480 (45.5)	
- Right	206 (45.6)	287 (47.5)	493 (46.7)	
- Bilateral	39 (8.6)	44 (7.3)	83 (7.9)	
Aspect ratio (>1 = 1/<1 = 0)				0.928
- 0	358 (79.2)	477 (79)	835 (79.1)	
- 1	94 (20.8)	127 (21)	221 (20.9)	
Spiculation				0.111
- No	139 (30.8)	214 (35.4)	353 (33.4)	
- Yes	313 (69.2)	390 (64.6)	703 (66.6)	
Blood flow				0.012
- No	155 (34.3)	253 (41.9)	408 (38.6)	
- Yes	297 (65.7)	351 (58.1)	648 (61.4)	
Axillary lymph nodes				0.362
- No	278 (61.5)	388 (64.2)	666 (63.1)	
- Yes	174 (38.5)	216 (35.8)	390 (36.9)	
Tumor location				0.214
- 0	0 (0)	7 (1.2)	7 (0.7)	
- Upper inner	93 (20.6)	116 (19.2)	209 (19.8)	
- Upper outer	275 (60.8)	362 (59.9)	637 (60.3)	
- Lower inner	27 (6)	41 (6.8)	68 (6.4)	
- Lower outer	57 (12.6)	78 (12.9)	135 (12.8)	
Biopsy (time)				0.641
- No	249 (55.1)	324 (53.6)	573 (54.3)	
- Yes	203 (44.9)	280 (46.4)	483 (45.7)	
Distant metastasis				0.157
- No	401 (88.7)	518 (85.8)	919 (87)	
- Yes	51 (11.3)	86 (14.2)	137 (13)	
Breast-conserving surgery				0.162
- No	279 (61.7)	398 (65.9)	677 (64.1)	
- Yes	173 (38.3)	206 (34.1)	379 (35.9)	
Lymph node dissection				<0.001
- No	362 (80.1)	532 (88.1)	894 (84.7)	
- Yes	90 (19.9)	72 (11.9)	162 (15.3)	
Pathological tumor diameter (cm), median (IQR)	2.5 (2,3.5)	1.5 (0.7,1.8)	1.8 (1.2,3)	<0.001
Histological grade				0.032
- 0	20 (4.4)	34 (5.6)	54 (5.1)	
- G1	65 (14.4)	92 (15.2)	157 (14.9)	
- G2	202 (44.7)	308 (51)	510 (48.3)	
- G3	148 (32.7)	144 (23.8)	292 (27.7)	
- G4	17 (3.8)	26 (4.3)	43 (4.1)	
Lymph node status, median (IQR)	0 (0,0.1)	0 (0,0.1)	0 (0,0.1)	0.274
Lymphovascular invasion				0.024
- No	335 (74.1)	483 (80)	818 (77.5)	
- Yes	117 (25.9)	121 (20)	238 (22.5)	
ER (%), median (IQR)	70 (0,90)	80 (5,90)	80 (4.5,90)	0.098
PR (%), median (IQR)	20 (0,80)	40 (0,80)	30 (0,80)	0.062
Ki-67 (%), median (IQR)	30 (20,60)	40 (20,60)	35 (20,60)	0.567
HER2				0.043
- 0	181 (40)	211 (34.9)	392 (37.1)	
- 1	98 (21.7)	109 (18)	207 (19.6)	
- 2	67 (14.8)	110 (18.2)	177 (16.8)	
- 3	106 (23.5)	174 (28.8)	280 (26.5)	
Adjuvant therapy				0.012
- No	49 (10.8)	98 (16.2)	147 (13.9)	
- Yes	403 (89.2)	506 (83.8)	909 (86.1)	
Palliative therapy				0.447
- No	428 (94.7)	578 (95.7)	1006 (95.3)	
- Yes	24 (5.3)	26 (4.3)	50 (4.7)	
Chemotherapy				0.034
- No	118 (26.1)	194 (32.1)	312 (29.5)	
- Yes	334 (73.9)	410 (67.9)	744 (70.5)	
Endocrine therapy				0.546
- No	209 (46.2)	268 (44.4)	477 (45.2)	
- Yes	243 (53.8)	336 (55.6)	579 (54.8)	
Targeted therapy				0.951
- No	318 (70.4)	426 (70.5)	744 (70.5)	
- Yes	134 (29.6)	178 (29.5)	312 (29.5)	
Immunotherapy				0.029
- No	433 (95.8)	559 (92.5)	992 (93.9)	
- Yes	19 (4.2)	45 (7.5)	64 (6.1)	
Pathological type				0.349
- Invasive ductal carcinoma	395 (87.4)	548 (90.7)	943 (89.3)	
- Invasive lobular carcinoma	9 (2)	9 (1.5)	18 (1.7)	
- Carcinoma in situ	24 (5.3)	21 (3.5)	45 (4.3)	
- Other	24 (5.3)	26 (4.3)	50 (4.7)	

### Predictor selection

Univariate cox regression analysis identified multiple significant predictors (**Table 3**). Multivariate Cox regression revealed that tumor size (cm) (HR = 1.025, 95%CI: 1.013-1.037, p<0.001), lymph node dissection (HR = 0.278, 95%CI: 0.199-0.389, p<0.001), ER% (HR = 1.006, 95%CI: 1.003-1.009, p<0.001), PR% (HR = 1.005, 95%CI: 1.002-1.008, p=0.002), Ki-67% (HR = 1.012, 95%CI: 1.007-1.016, p<0.001), and HER2 status (HR = 1.195, 95%CI: 1.098-1.301, p<0.001) were independent prognostic factors for breast cancer DFS (**Table 4**). These six variables were subsequently used as the uniform input features for all seven machine learning models. A supplementary LASSO-based selection yielded the same core variables plus SII, but the discriminatory gain was negligible (C-index increase <0.01), supporting our use of the six parsimonious predictors.

**Table 3:** Univariate cox regression analysis.

Variable	B	SE	Wald	df	Sig.	Exp(B)	95% CI for Exp(B)	
							Lower	Upper
Age	0.001	0.005	0.03	1	0.863	1.001	0.992	1.01
Diabetes	0.382	0.236	2.626	1	0.105	1.465	0.923	2.325
Hyperlipidemia	0.389	0.248	2.463	1	0.117	1.476	0.908	2.4
Hypertension	0.065	0.184	0.122	1	0.726	1.067	0.743	1.531
BMI	0.005	0.016	0.115	1	0.735	1.006	0.974	1.038
Prior tumor	0.236	0.167	1.997	1	0.158	1.266	0.913	1.756
BI-RADS	0.04	0.041	0.954	1	0.329	1.041	0.96	1.129
Size (cm)	0.025	0.006	18.386	1	<0.001	1.025	1.014	1.037
Laterality (Right)			1.338	2	0.512			
Bilateral	-0.113	0.103	1.198	1	0.274	0.893	0.73	1.093
Aspect ratio	0.015	0.203	0.005	1	0.941	1.015	0.681	1.512
Spiculation	-0.112	0.123	0.832	1	0.362	0.894	0.703	1.137
Blood flow	-0.165	0.104	2.507	1	0.113	0.848	0.691	1.04
Axillary LN	0.011	0.101	0.011	1	0.915	1.011	0.829	1.232
Tumor location	-0.107	0.103	1.072	1	0.3	0.899	0.734	1.1
Location (Upper inner)			4.057	4	0.398			
Upper outer	-0.634	0.462	1.887	1	0.169	0.53	0.215	1.311
Lower inner	-0.634	0.452	1.961	1	0.161	0.531	0.219	1.288
Lower outer	-0.417	0.482	0.749	1	0.387	0.659	0.257	1.694
Biopsy	-0.469	0.469	1.001	1	0.317	0.626	0.25	1.568
Distant metastasis	0.031	0.099	0.096	1	0.757	1.031	0.849	1.253
Breast conservation	0.024	0.143	0.028	1	0.867	1.024	0.774	1.356
LN dissection	-0.123	0.105	1.357	1	0.244	0.884	0.719	1.087
Pathological tumor diameter	-1.349	0.169	63.781	1	<0.001	0.259	0.186	0.361
Histological grade (G1)	-0.042	0.044	0.923	1	0.337	0.959	0.879	1.045
Grade (G2)	-0.098	0.057	2.989	1	0.084	0.906	0.811	1.013
Grade (G3)	-0.098	0.057	2.989	1	0.084	0.906	0.811	1.013
LN status	-0.179	0.179	0.998	1	0.318	0.836	0.588	1.188
LVI	0.048	0.12	0.158	1	0.691	1.049	0.829	1.327
ER (%)	0.007	0.001	30.262	1	<0.001	1.007	1.005	1.01
PR (%)	0.006	0.001	21.529	1	<0.001	1.006	1.004	1.009
Ki-67 (%)	0.01	0.002	19.343	1	<0.001	1.01	1.005	1.014
HER2	0.202	0.041	24.027	1	<0.001	1.224	1.129	1.326
Adjuvant therapy	-0.178	0.138	1.661	1	0.198	0.837	0.638	1.097
Palliative therapy	-0.196	0.233	0.708	1	0.4	0.822	0.521	1.297
Chemotherapy	-0.15	0.107	1.969	1	0.161	0.861	0.698	1.061
Endocrine therapy	0.072	0.099	0.518	1	0.472	1.074	0.884	1.305
Targeted therapy	-0.173	0.11	2.456	1	0.117	0.841	0.678	1.044
Immunotherapy	0.108	0.19	0.324	1	0.569	1.114	0.768	1.617
Pathological type			0.984	3	0.805			
Invasive lobular	-0.176	0.503	0.122	1	0.727	0.839	0.313	2.248
Carcinoma in situ	-0.255	0.293	0.754	1	0.385	0.775	0.436	1.378
Other	-0.091	0.235	0.148	1	0.7	0.913	0.576	1.449

**Table 4:** Multivariate cox regression analysis.

Variable	B	SE	df	Sig.	Exp(B)	95% CI for Exp(B)	
						Lower	Upper
Size (cm)	0.025	0.006	1	<0.001	1.025	1.013	1.037
Lymph node dissection	-1.279	0.171	1	<0.001	0.278	0.199	0.389
ER (%)	0.006	0.002	1	<0.001	1.006	1.003	1.009
PR (%)	0.005	0.002	1	0.002	1.005	1.002	1.008
Ki-67 (%)	0.011	0.002	1	<0.001	1.012	1.007	1.016
HER2	0.178	0.043	1	<0.001	1.195	1.098	1.301

### Comparison of multiple models

Model performance was evaluated using time-dependent AUC and Brier scores for 1-year, 3-year, and 5-year predictions in both training and validation sets. 95% confidence intervals for key metrics and the Concordance index (C-index) are provided in **Table 5**. Random Forest achieved the highest time-dependent AUC values across all time points: 0.924 (95%CI: 0.891-0.957, 1-year), 0.890 (95%CI: 0.848-0.932, 3-year), and 0.952 (95%CI: 0.923-0.981, 5-year) in the training set. XGBoost demonstrated excellent performance with AUCs of 0.889 (95%CI: 0.848-0.930, 1-year), 0.866 (95%CI: 0.821-0.911, 3-year), and 0.971 (95%CI: 0.948-0.994, 5-year), showing superior long-term predictive capacity. The Cox model showed moderate performance with AUCs ranging from 0.721 to 0.856.

**Table 5:** Comparison of model performance metrics.

Metric		Cox	LASSO	Elastic-Net	Decision tree	Random forest	XGBoost	GBM
Training Set	AUC (1-year)	0.806	0.793	0.783	0.736	0.924	0.889	0.875
	AUC (3-year)	0.721	0.726	0.730	0.705	0.890	0.866	0.840
	AUC (5-year)	0.856	0.859	0.859	0.827	0.952	0.971	0.953
	Brier (1-year)	0.109	0.125	0.131	0.106	0.078	0.073	0.109
	Brier (3-year)	0.210	0.218	0.238	0.209	0.154	0.148	0.193
	Brier (5-year)	0.128	0.143	0.183	0.131	0.089	0.070	0.093
Validation Set	AUC (1-year)	0.824	0.808	0.765	0.753	0.851	0.779	0.791
	AUC (3-year)	0.772	0.782	0.785	0.750	0.763	0.751	0.766
	AUC (5-year)	0.803	0.789	0.803	0.822	0.826	0.804	0.805
	Brier (1-year)	0.107	0.133	0.142	0.098	0.089	0.104	0.122
	Brier (3-year)	0.193	0.205	0.237	0.201	0.197	0.206	0.205
	Brier (5-year)	0.151	0.157	0.194	0.140	0.142	0.155	0.159

For Brier scores, Random Forest (1-year: 0.078, 3-year: 0.154, 5-year: 0.089) and XGBoost (1-year: 0.073, 3-year: 0.148, 5-year: 0.070) demonstrated the lowest scores, indicating superior calibration. In contrast, Decision Tree showed higher Brier scores across all time points, suggesting poorer calibration.

In the validation set, Random Forest maintained robust performance with 1-year AUC of 0.851 (95%CI: 0.802-0.900), 3-year AUC of 0.763 (95%CI: 0.702-0.824), and 5-year AUC of 0.826 (95%CI: 0.766-0.886). The C-index for Random Forest in the validation set was 0.803 (95%CI: 0.762-0.844), confirming robust overall discriminative capacity. XGBoost showed strong 5-year predictive capacity (AUC = 0.804, 95%CI: 0.740-0.868) in the validation set. For Brier scores in validation, Random Forest achieved the lowest scores at 1-year (0.089) and 3-year (0.197), indicating good calibration. At 5-year, Decision Tree showed the lowest Brier score (0.140), followed by Random Forest (0.142).

Calibration metrics were consistently strong for Random Forest: in the validation set, calibration-in-the-large values were close to 0 and calibration slopes near 1 across all time points, confirming that predictions were neither systematically over- nor underestimated. Decision curve analysis demonstrated that Random Forest and XGBoost provided a net benefit over a wide range of clinically relevant threshold probabilities (approximately 10–50% for 5-year DFS), supporting their potential clinical value beyond simple discrimination.

Overall, Random Forest and XGBoost consistently outperformed other models in both discrimination and calibration, demonstrating significant advantages for short-term and long-term prediction. A detailed breakdown of performance metrics for all models is provided in **Table 5**.

### Cox model development and evaluation

**Figure 1:**
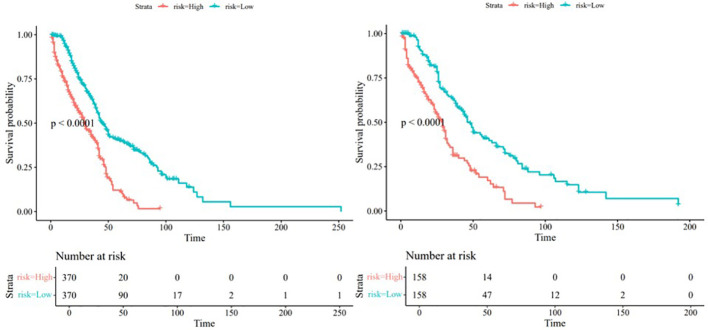
Kaplan-meier survival curves for high-risk vs. low-risk groups (Cox model).

**Figure 2:**
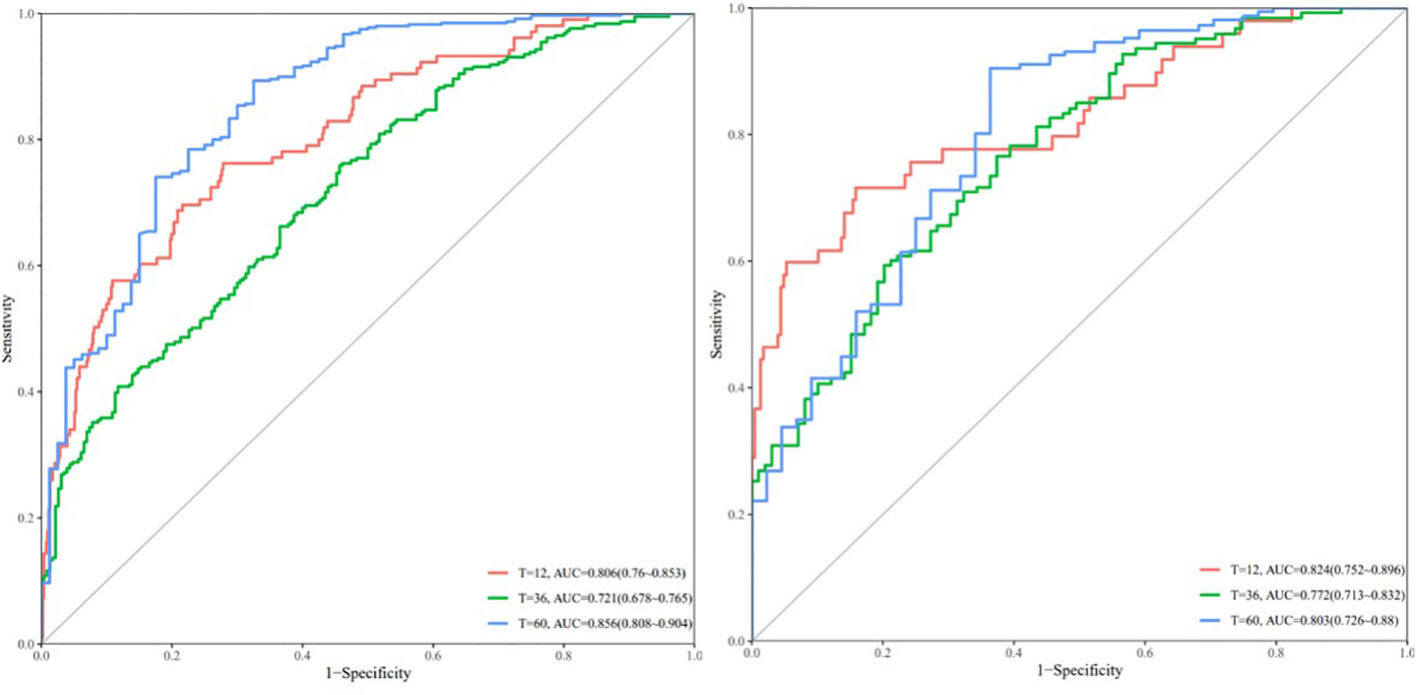
Time-dependent ROC curves for 1-year, 3-year, and 5-year predictions (Cox model).

**Figure 3:**
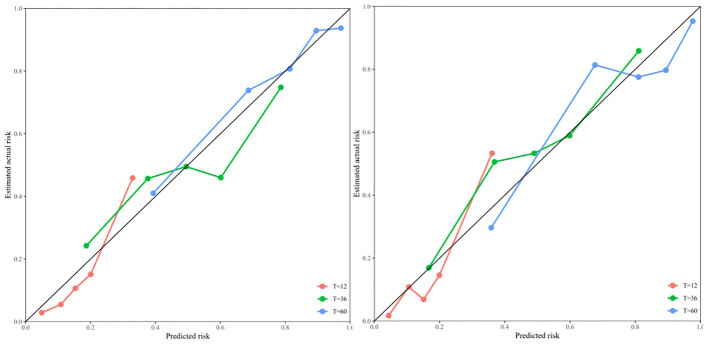
Calibration curves for 1-year, 3-year, and 5-year predictions (Cox model).

**Figure 4:**
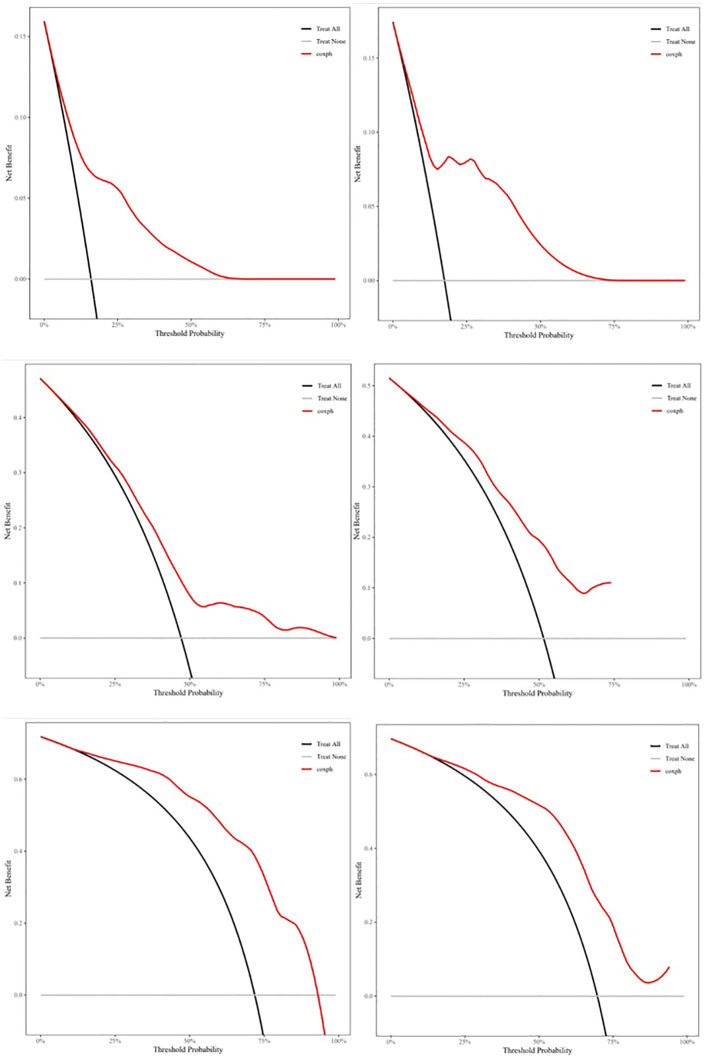
Decision curve analysis for 1-year, 3-year, and 5-year predictions (Cox model).

**Figure 5:**
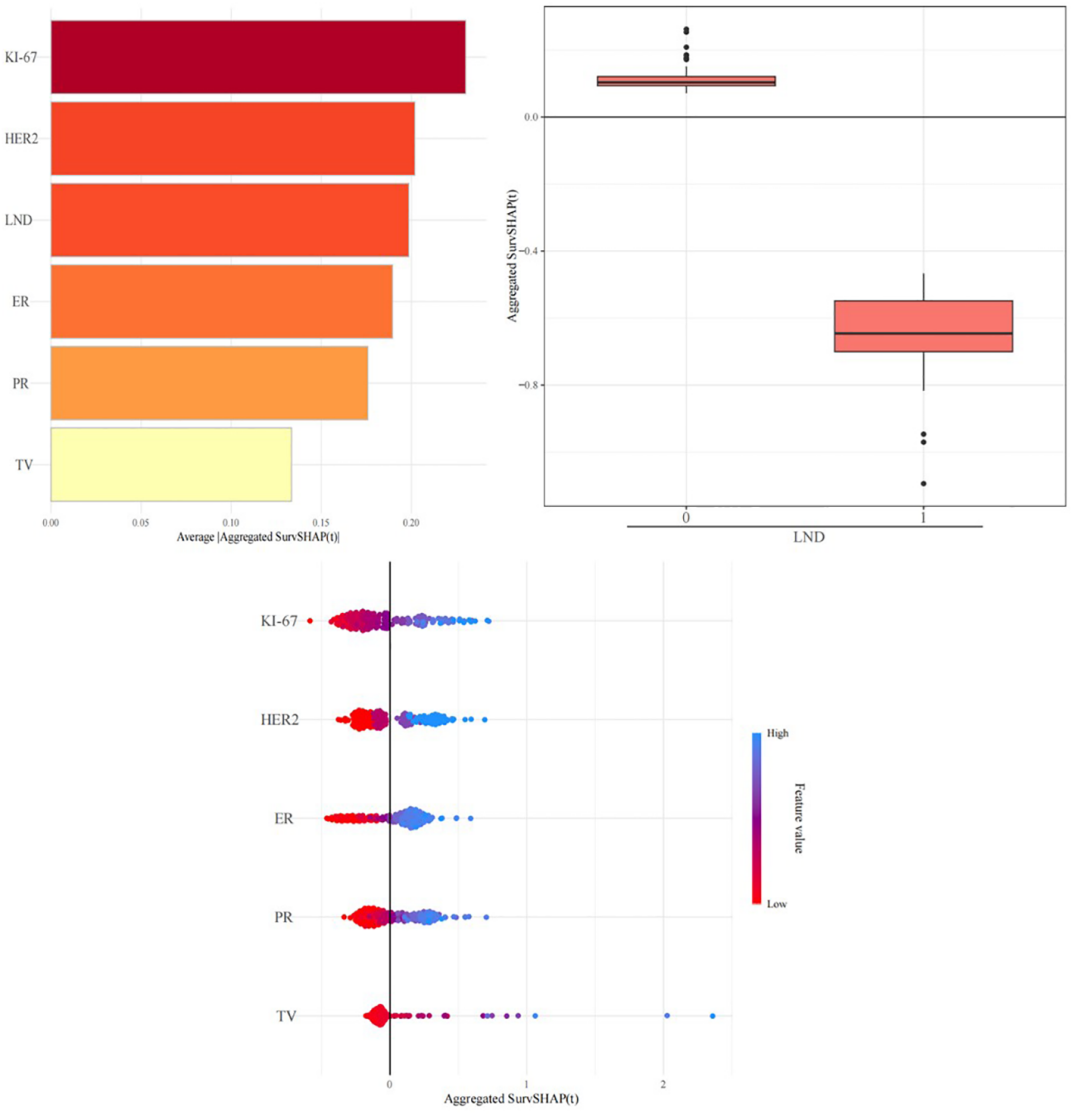
SHAP value distribution (Cox model).

Kaplan-Meier survival analysis comparing high-risk and low-risk groups demonstrated significantly higher survival probability in the low-risk group in both training (p<0.0001) and validation (p<0.0001) sets (**Figure 1**). The Cox proportional hazards model showed good predictive performance with time-dependent AUCs of 0.806 (1-year), 0.721 (3-year), and 0.856 (5-year) in the training set, and 0.824 (1-year), 0.772 (3-year), and 0.803 (5-year) in the validation set (**Figure 2**). Calibration curves (**Figure 3**) and decision curve analysis (**Figure 4**) confirmed potential clinical utility across different threshold probabilities, though net benefit was modest compared to ensemble methods. SHAP value distribution for the Cox model is shown in **Figure 5**.

### GBM model development and evaluation

**Figure 6:**
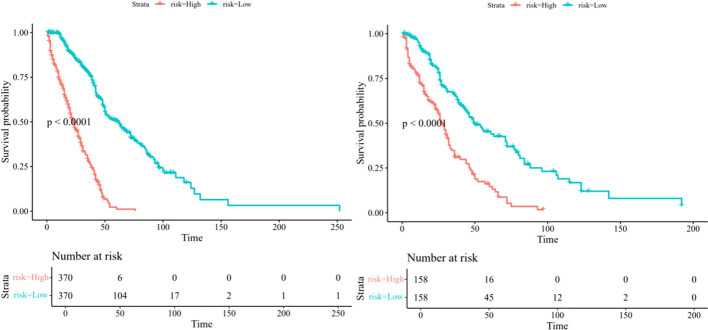
Kaplan-Meier survival curves (GBM model).

**Figure 7:**
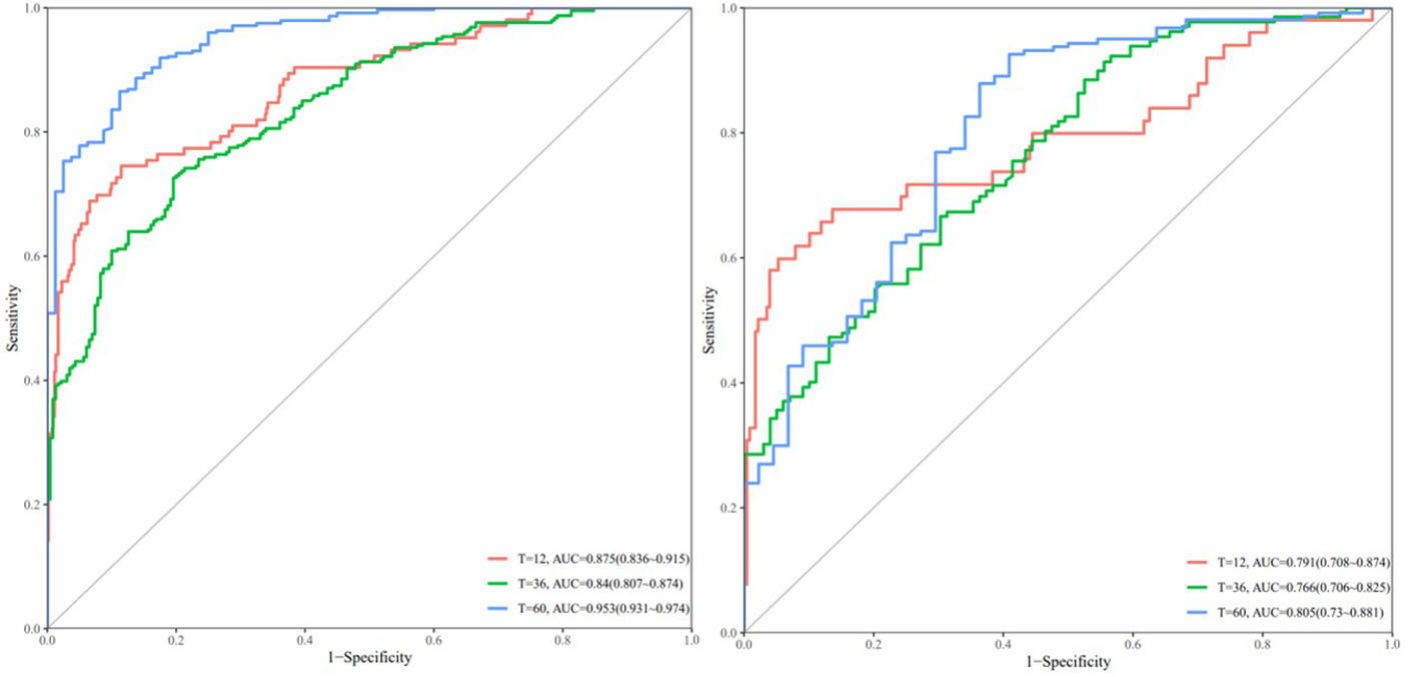
Time-dependent ROC curves (GBM model).

**Figure 8:**
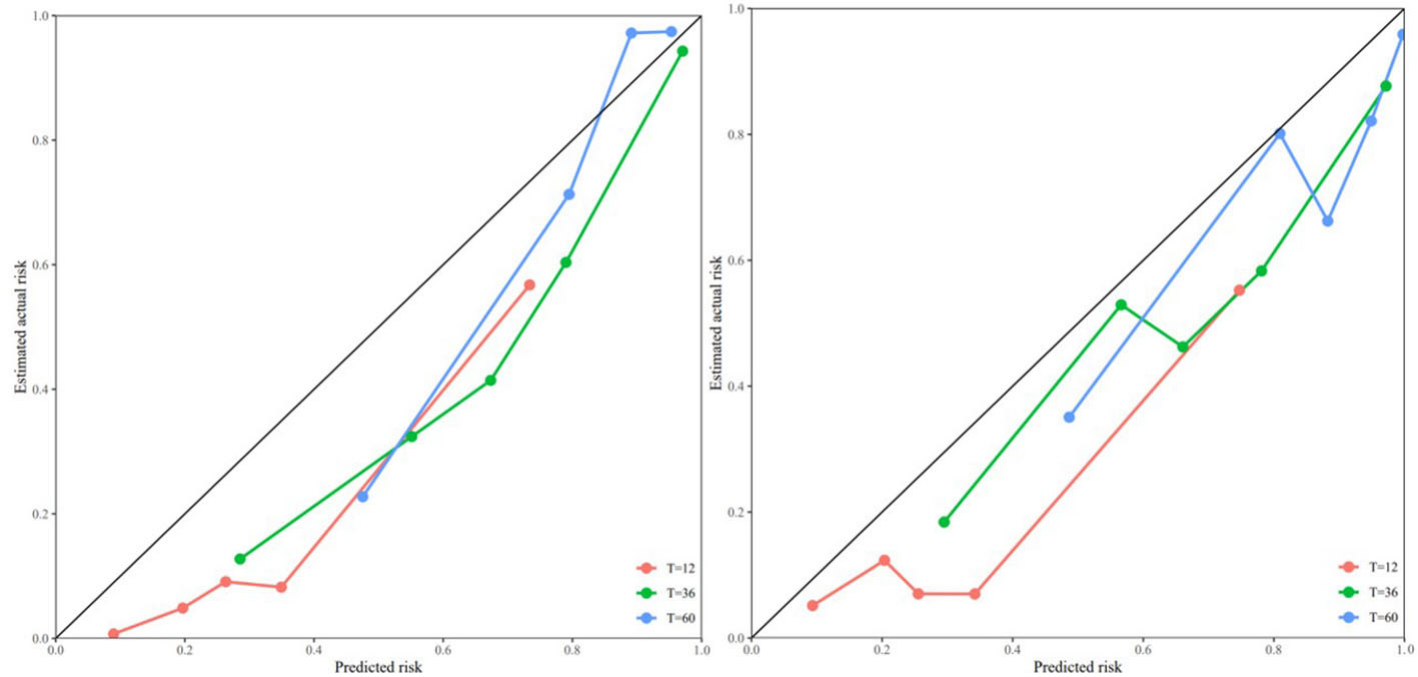
Calibration curves (GBM Model).

**Figure 9:**
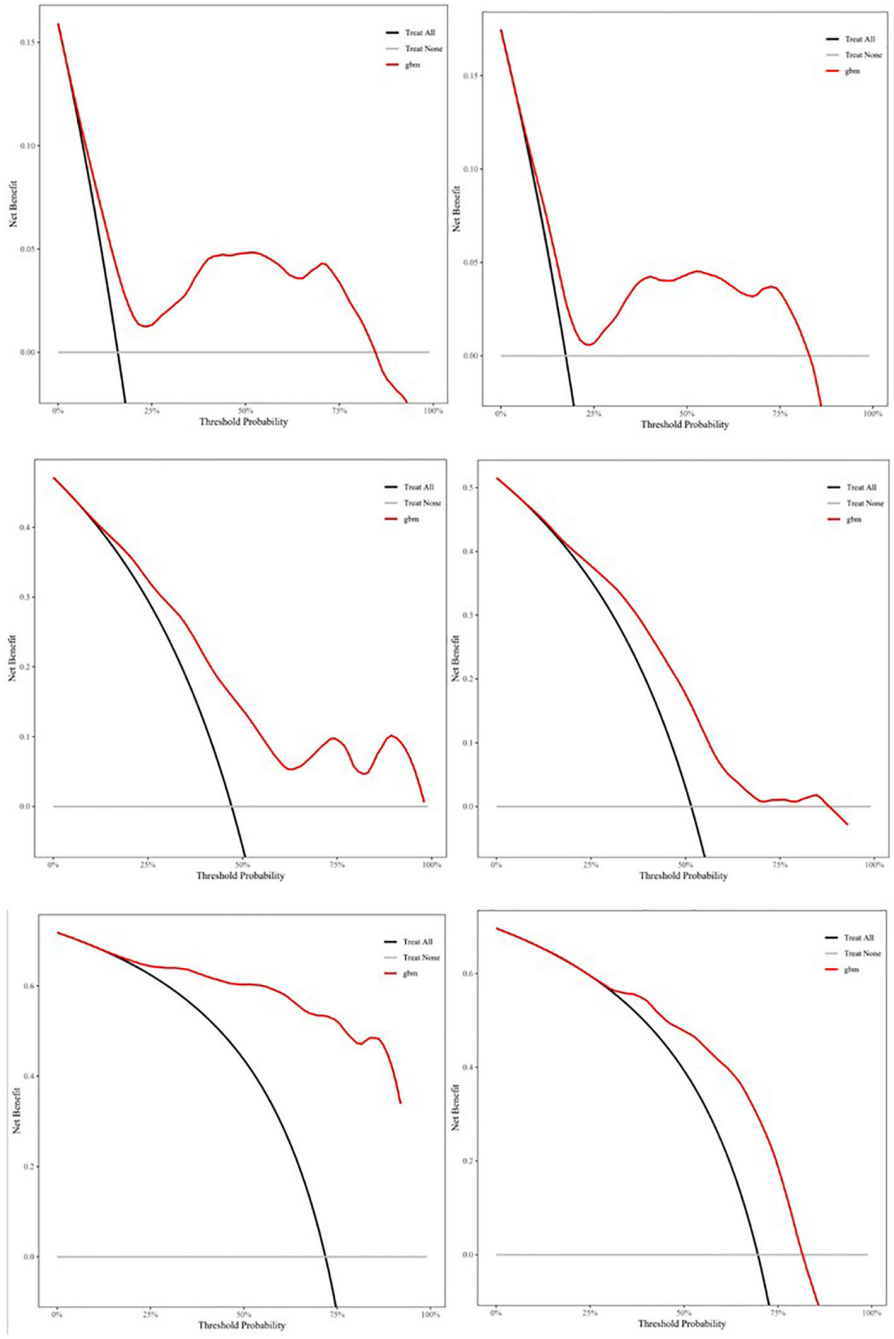
Decision curve analysis (GBM model).

**Figure 10:**
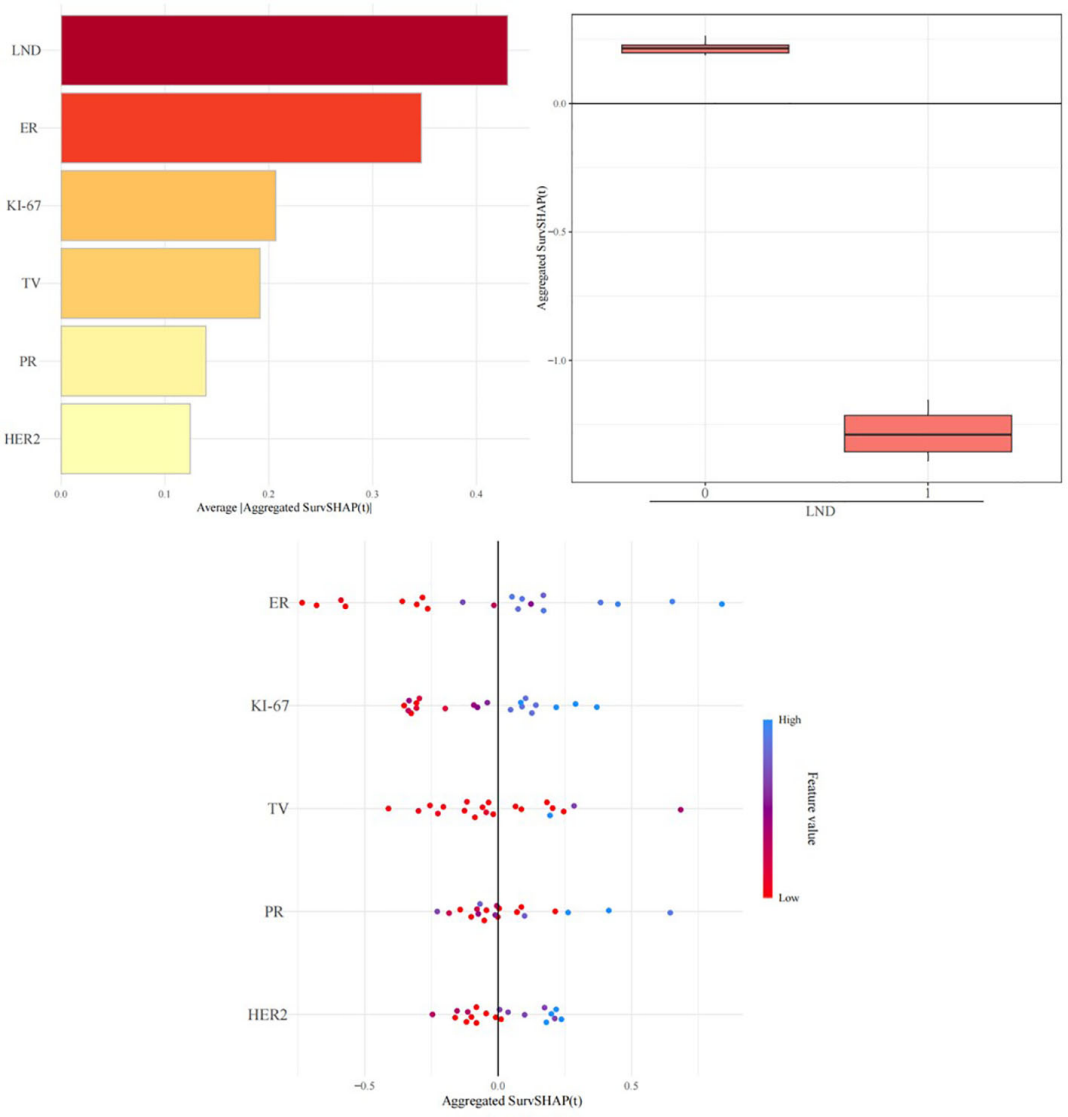
SHAP value distribution (GBM model).

Kaplan-Meier analysis revealed significant survival differences between risk groups (p<0.0001) (**Figure 6**). The GBM model demonstrated excellent discrimination with training set time-dependent AUCs of 0.875 (1-year), 0.840 (3-year), and 0.953 (5-year) (**Figure 7**). Validation set performance remained stable with AUCs of 0.791 (1-year), 0.766 (3-year), and 0.805 (5-year). Calibration curves showed good agreement between predicted and observed outcomes (**Figure 8**). Decision curve analysis (**Figure 9**) and SHAP value distribution (**Figure 10**) are provided.

### LASSO model development and evaluation

**Figure 11:**
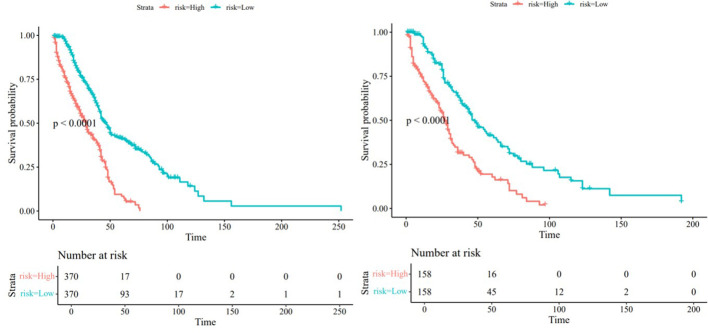
Kaplan-Meier survival curves (LASSO model).

**Figure 12:**
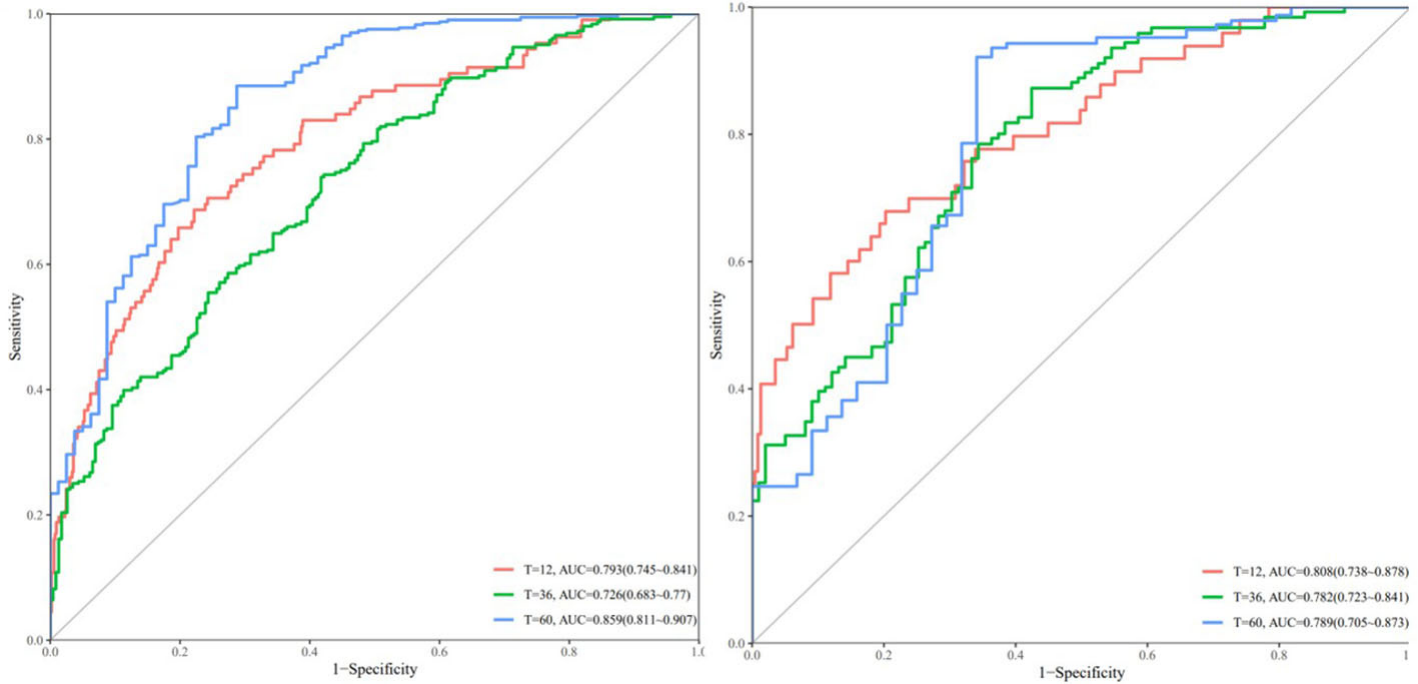
Time-dependent ROC curves (LASSO model).

**Figure 13:**
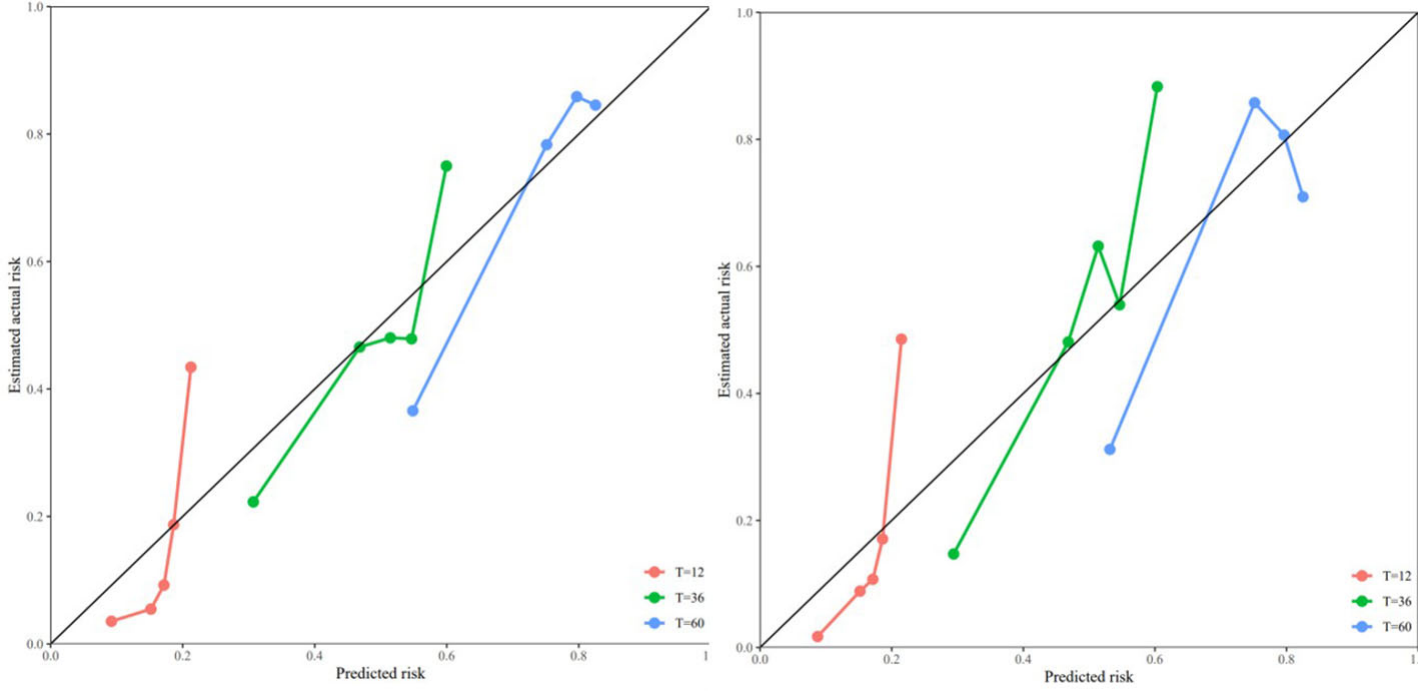
Calibration curves (LASSO model).

**Figure 14:**
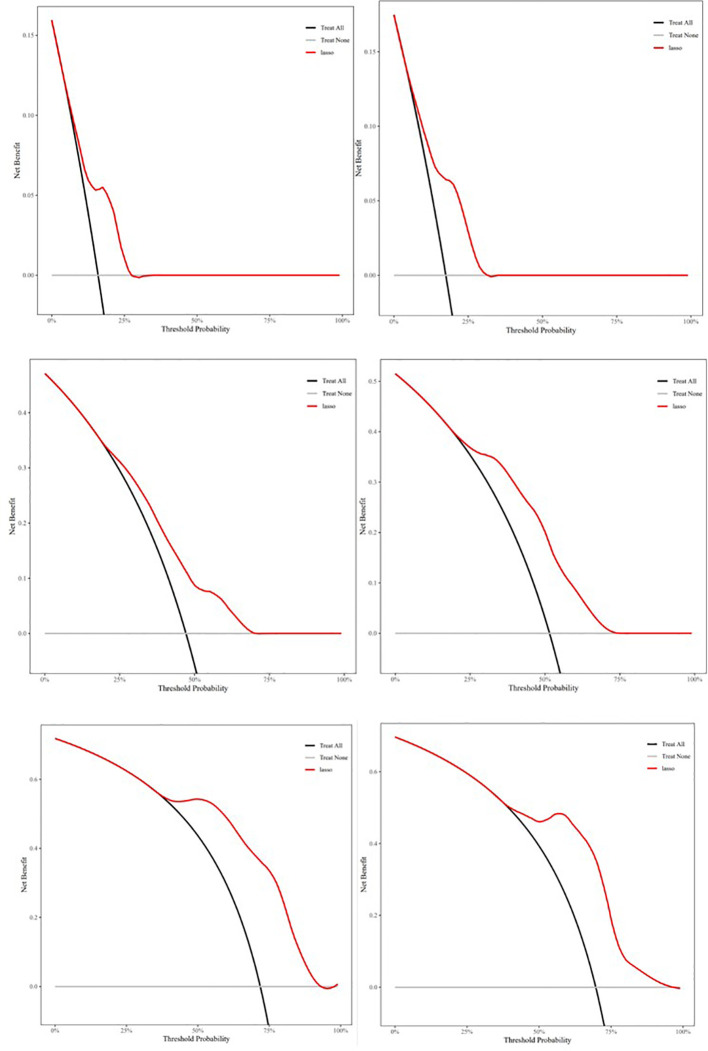
Decision curve analysis (LASSO model).

**Figure 15:**
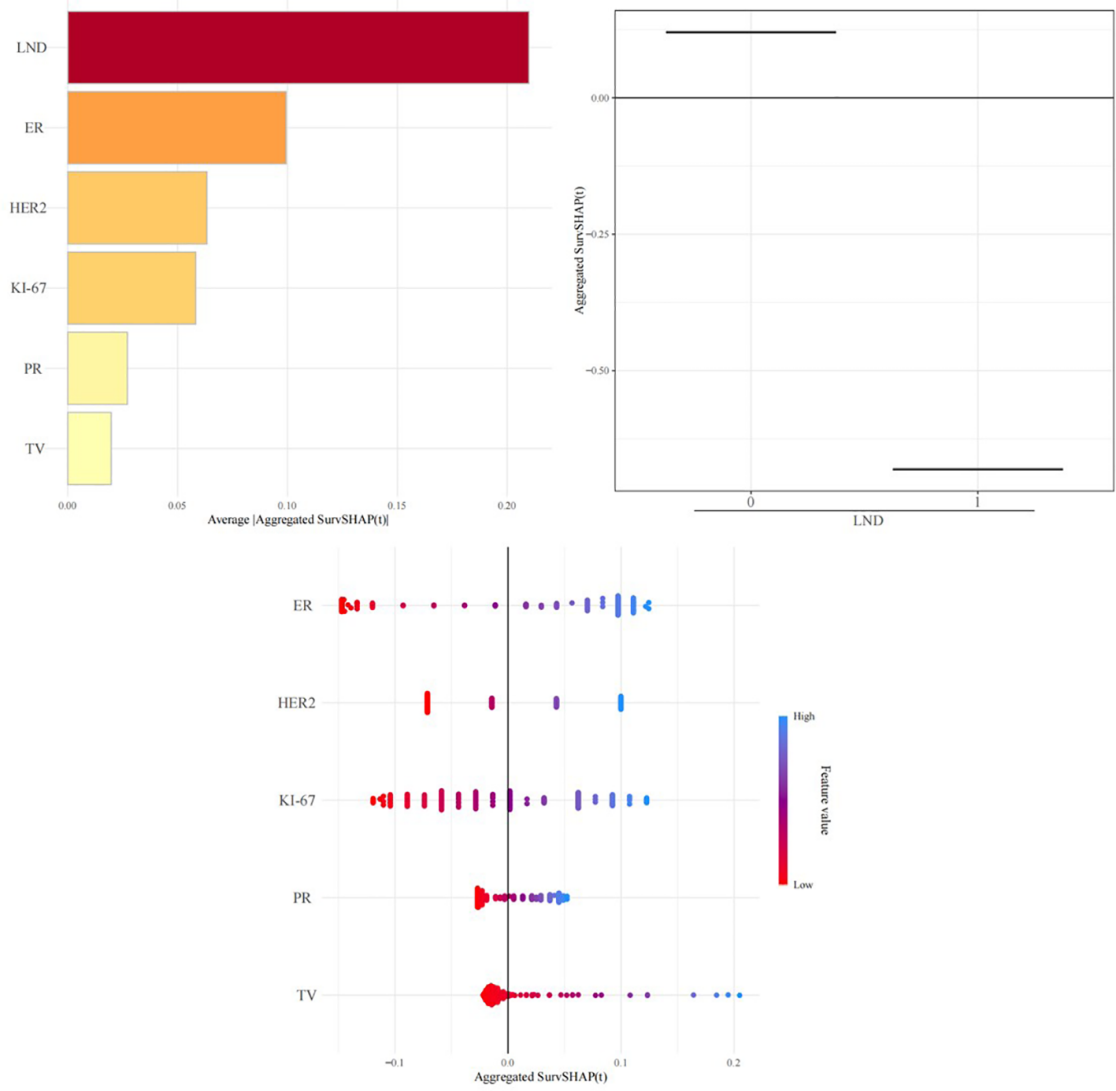
SHAP value distribution (LASSO model).

Kaplan-Meier analysis showed significant stratification between risk groups (p<0.0001) (**Figure 11**). The LASSO model achieved time-dependent AUCs of 0.793 (1-year), 0.726 (3-year), and 0.859 (5-year) in the training set (**Figure 12**). Validation set performance was comparable with AUCs of 0.808 (1-year), 0.782 (3-year), and 0.789 (5-year), demonstrating stable discrimination across time points. Calibration curves (**Figure 13**), decision curve analysis (**Figure 14**), and SHAP values (**Figure 15**) are presented.

### XGBoost model development and evaluation

**Figure 16:**
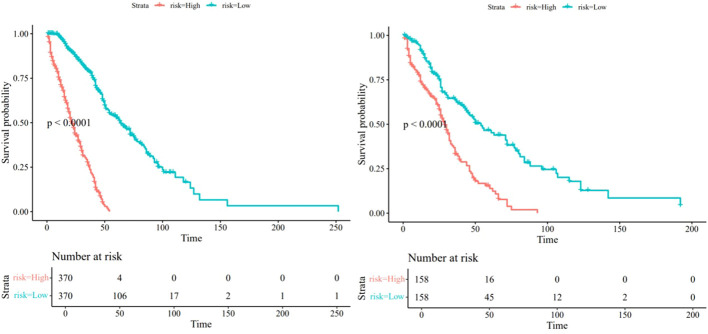
Kaplan-Meier survival curves (XGBoost model).

**Figure 17:**
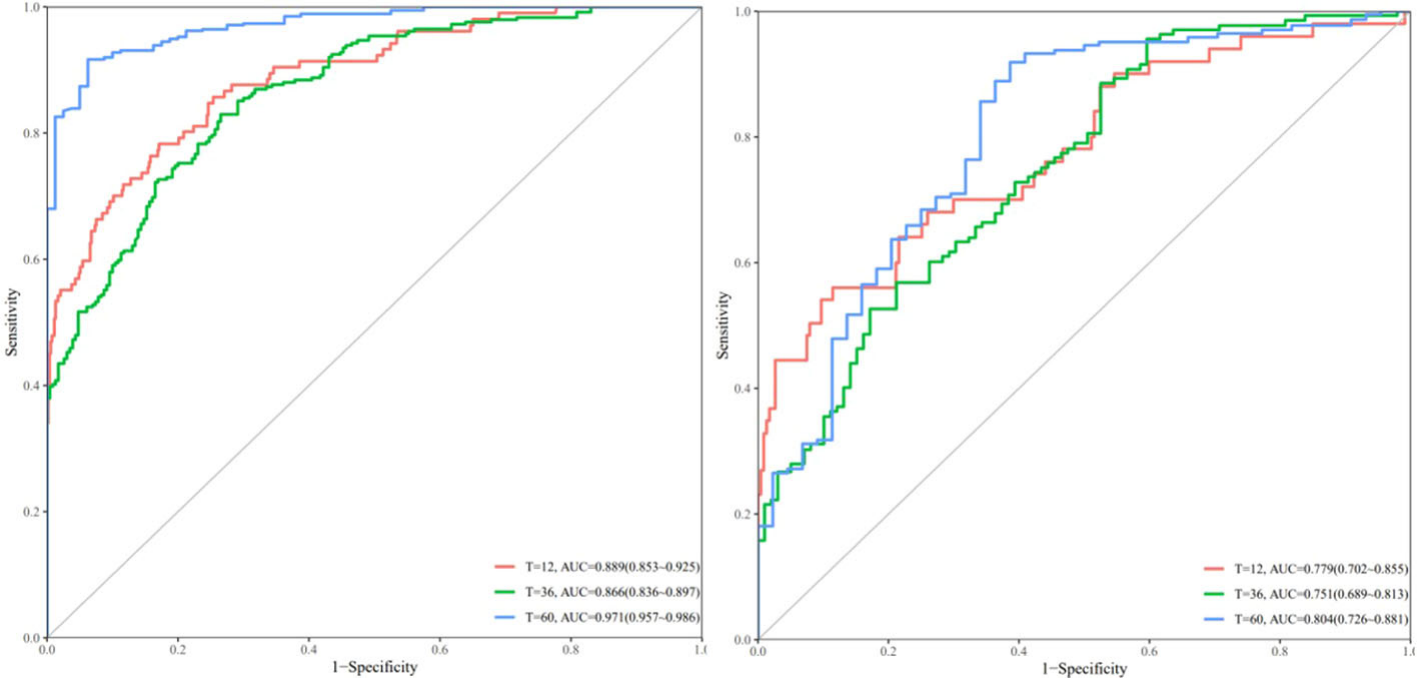
Time-dependent ROC curves (XGBoost model).

**Figure 18:**
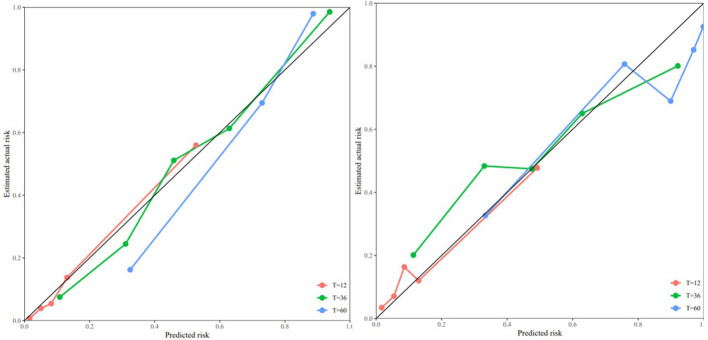
Calibration curves (XGBoost model).

**Figure 19:**
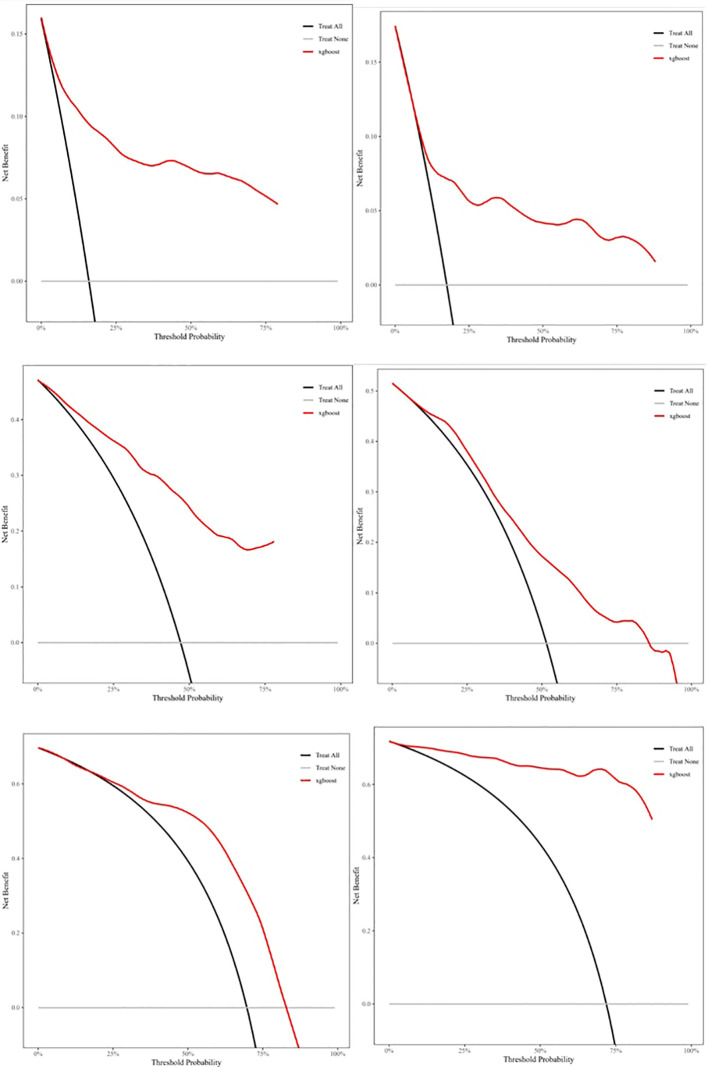
Decision curve analysis (XGBoost model).

**Figure 20:**
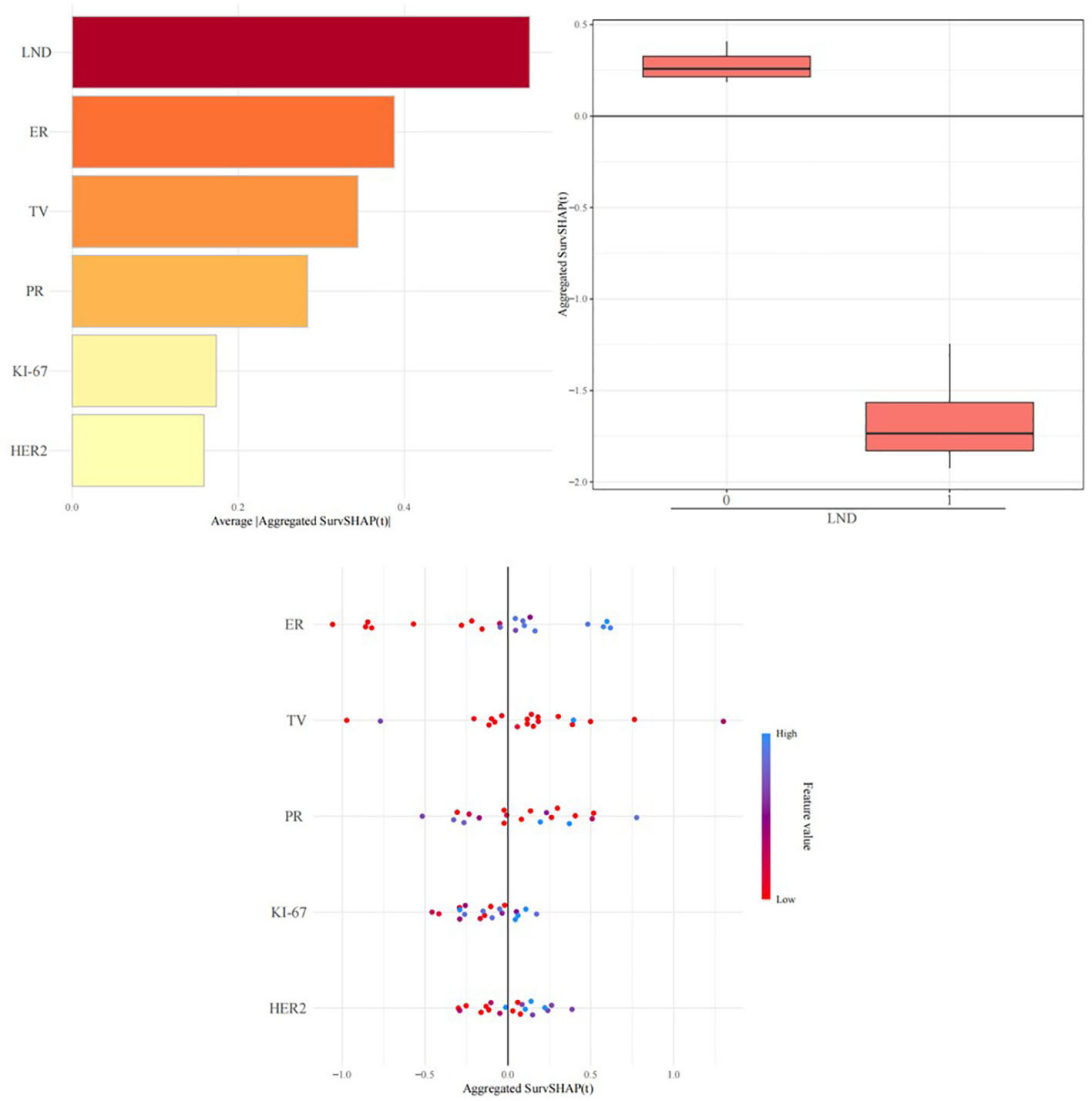
SHAP value distribution (XGBoost model).

Significant survival differences between risk groups were confirmed by Kaplan-Meier analysis (p<0.0001) (**Figure 16**). The XGBoost model demonstrated excellent predictive performance with training set time-dependent AUCs of 0.889 (1-year), 0.866 (3-year), and 0.971 (5-year) (**Figure 17**). Validation set AUCs were 0.779 (1-year), 0.751 (3-year), and 0.804 (5-year), indicating good generalization. Calibration curves (**Figure 18**), decision curve analysis (**Figure 19**), and SHAP value distribution (**Figure 20**) are shown.

### Elastic-net model development and evaluation

**Figure 21:**
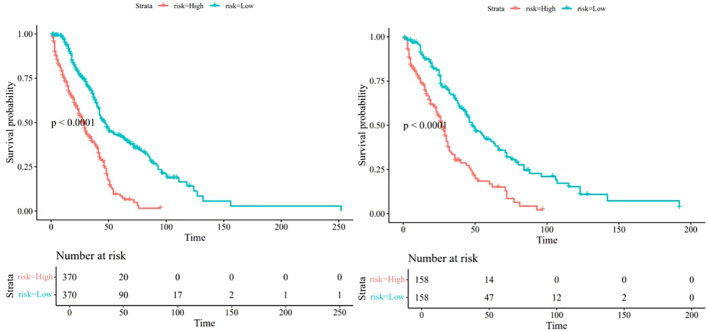
Kaplan-Meier survival curves (elastic-net model).

**Figure 22:**
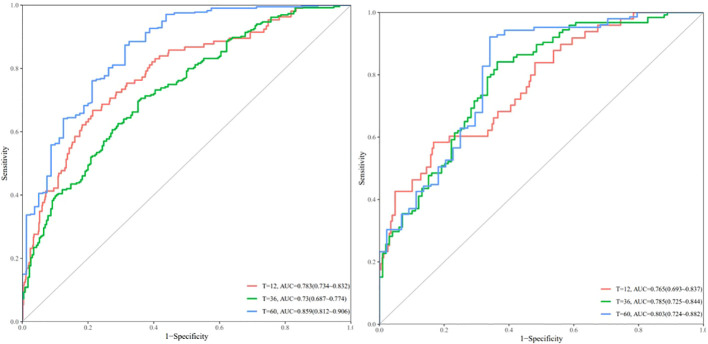
Time-dependent ROC curves (elastic-net model).

**Figure 23:**
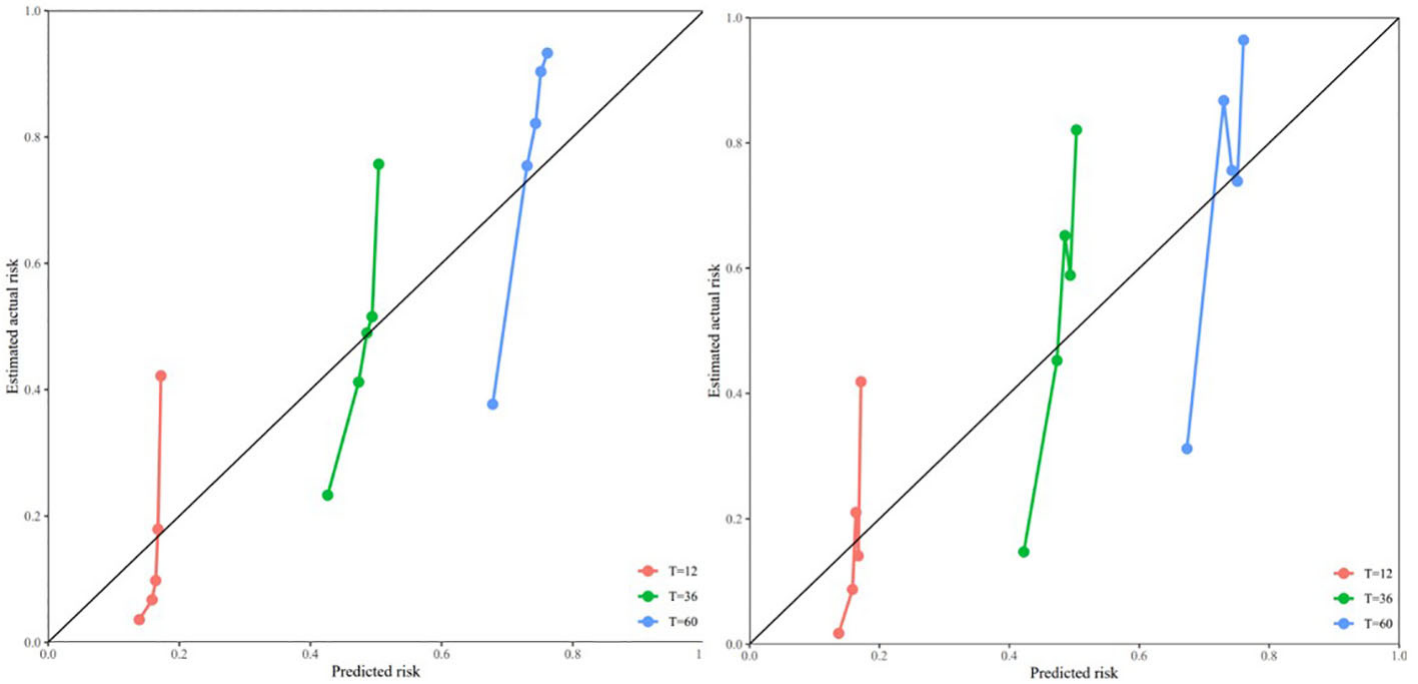
Calibration curves (elastic-net model).

**Figure 24:**
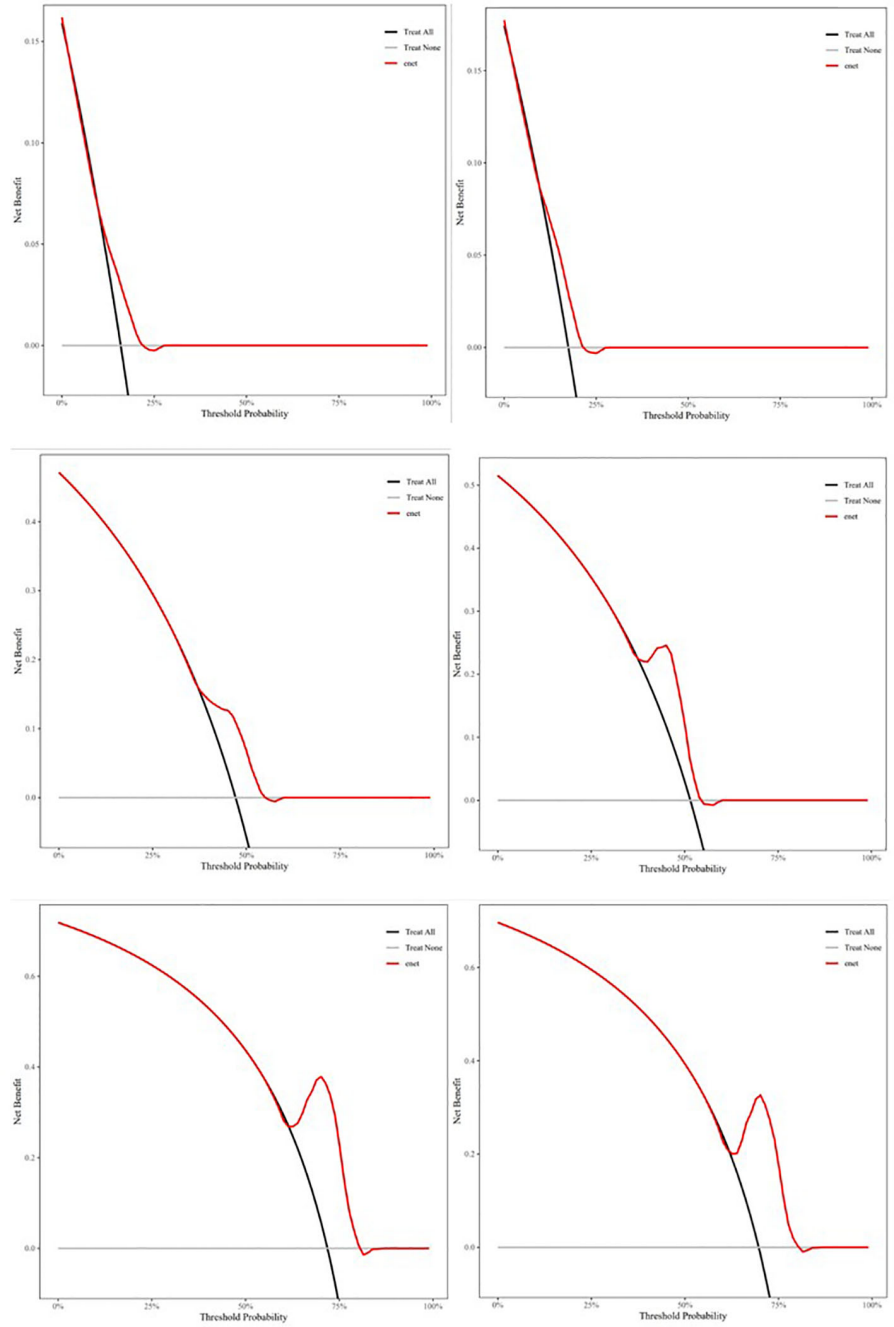
Decision curve analysis (elastic-net model).

**Figure 25:**
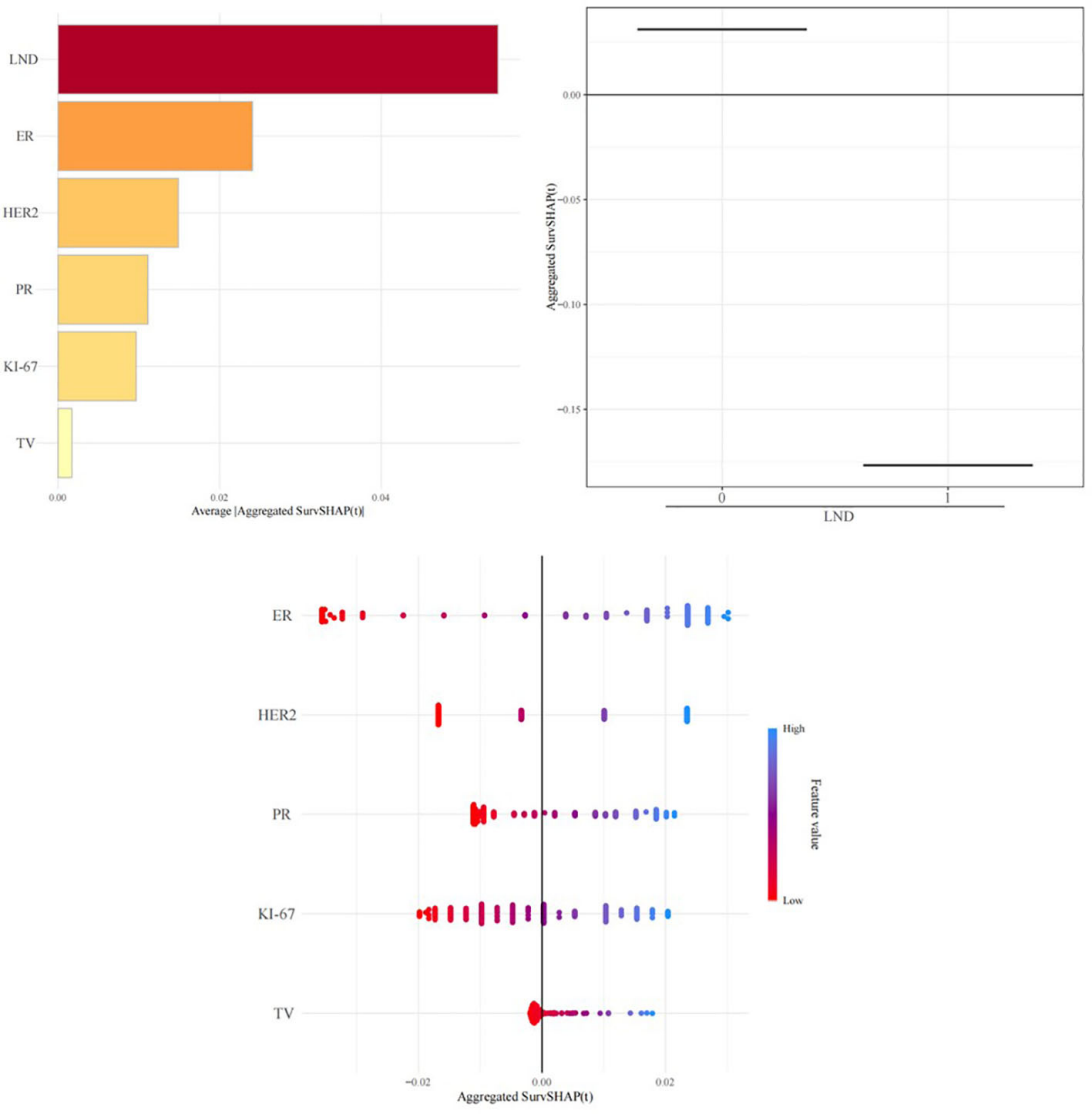
SHAP Value distribution (elastic-net model).

Kaplan-Meier analysis revealed significant risk stratification (p<0.0001) (**Figure 21**). The Elastic-Net model achieved training set time-dependent AUCs of 0.783 (1-year), 0.730 (3-year), and 0.859 (5-year) (**Figure 22**). Validation set performance was robust with AUCs of 0.765 (1-year), 0.785 (3-year), and 0.803 (5-year), demonstrating stable long-term predictive capacity. Calibration curves (**Figure 23**), decision curve analysis (**Figure 24**), and SHAP values (**Figure 25**) are provided.

### Decision tree model development and evaluation

**Figure 26:**
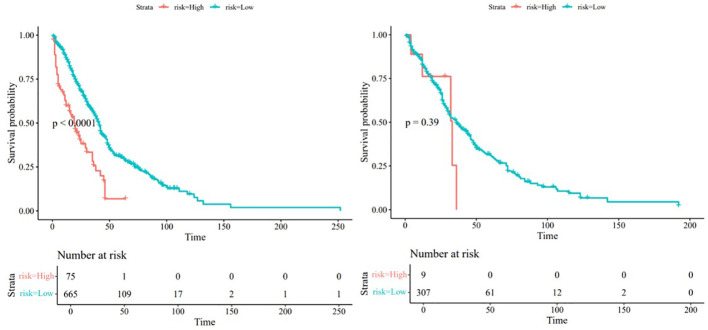
Kaplan-Meier Survival curves (decision tree model).

**Figure 27:**
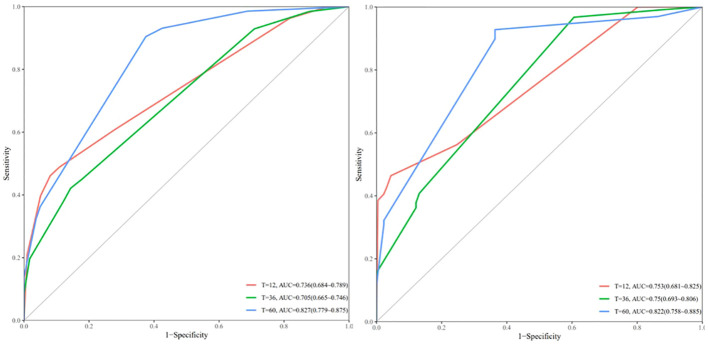
Time-dependent ROC curves (decision tree model).

**Figure 28:**
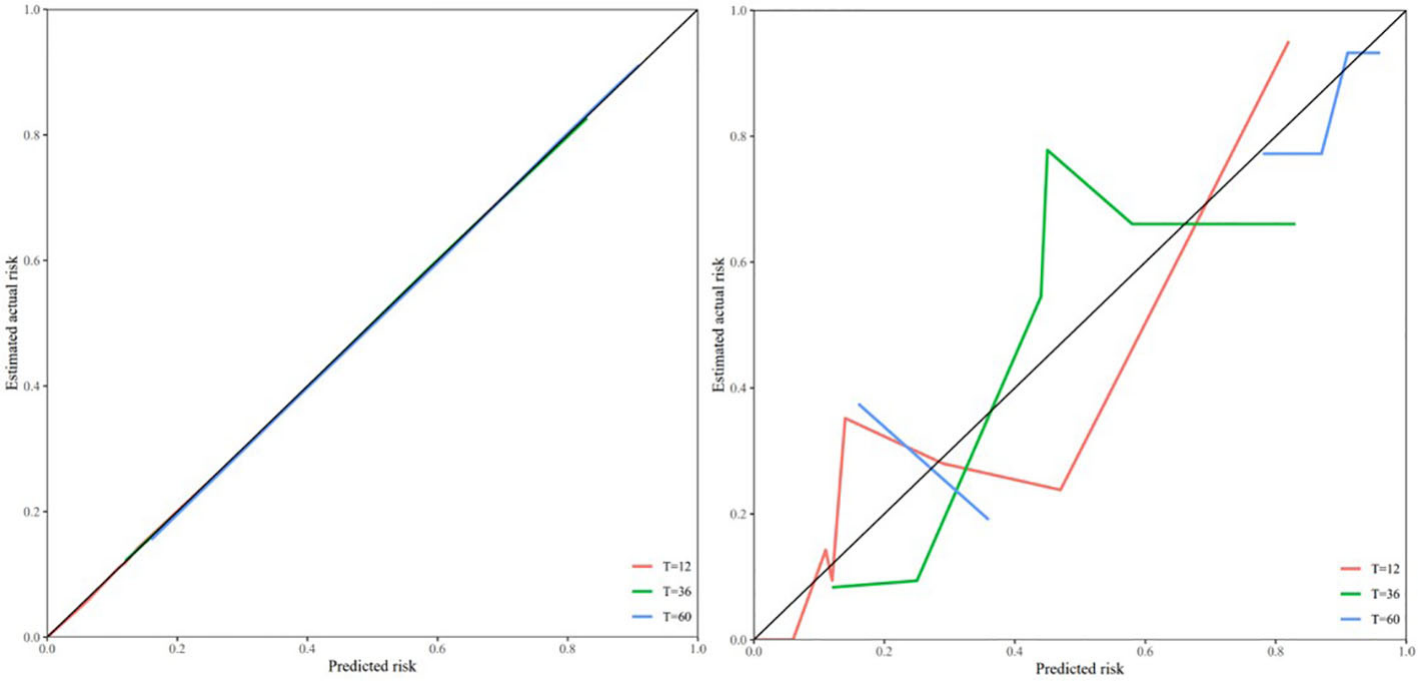
Calibration curves (decision tree model).

**Figure 29:**
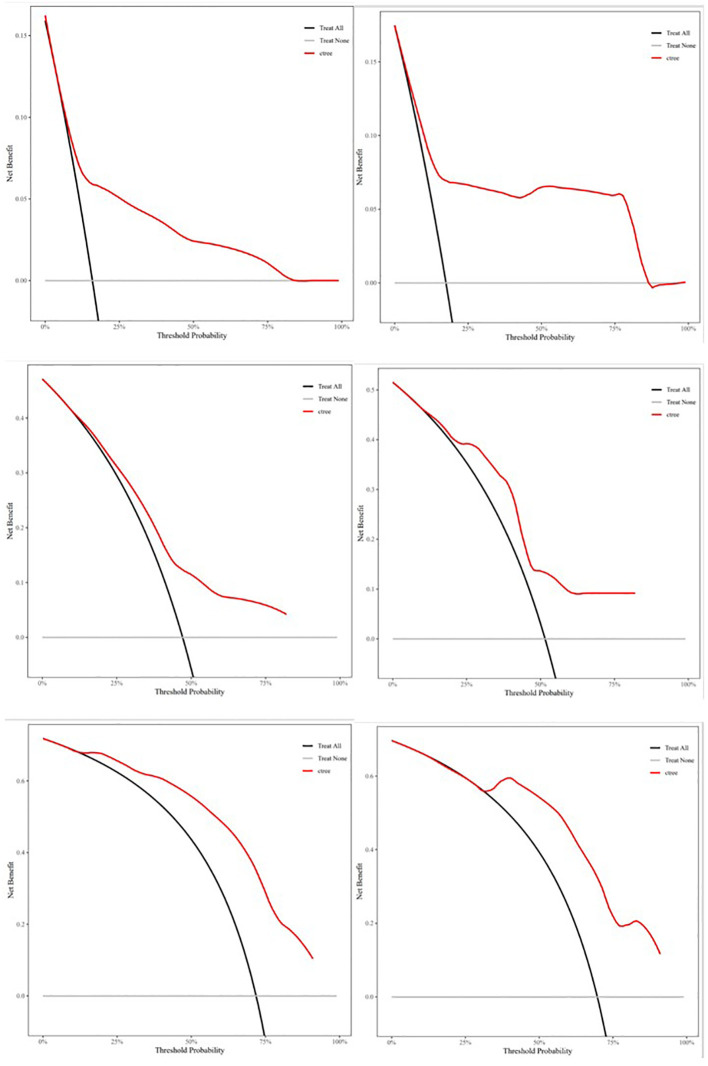
Decision curve analysis (decision tree model).

**Figure 30:**
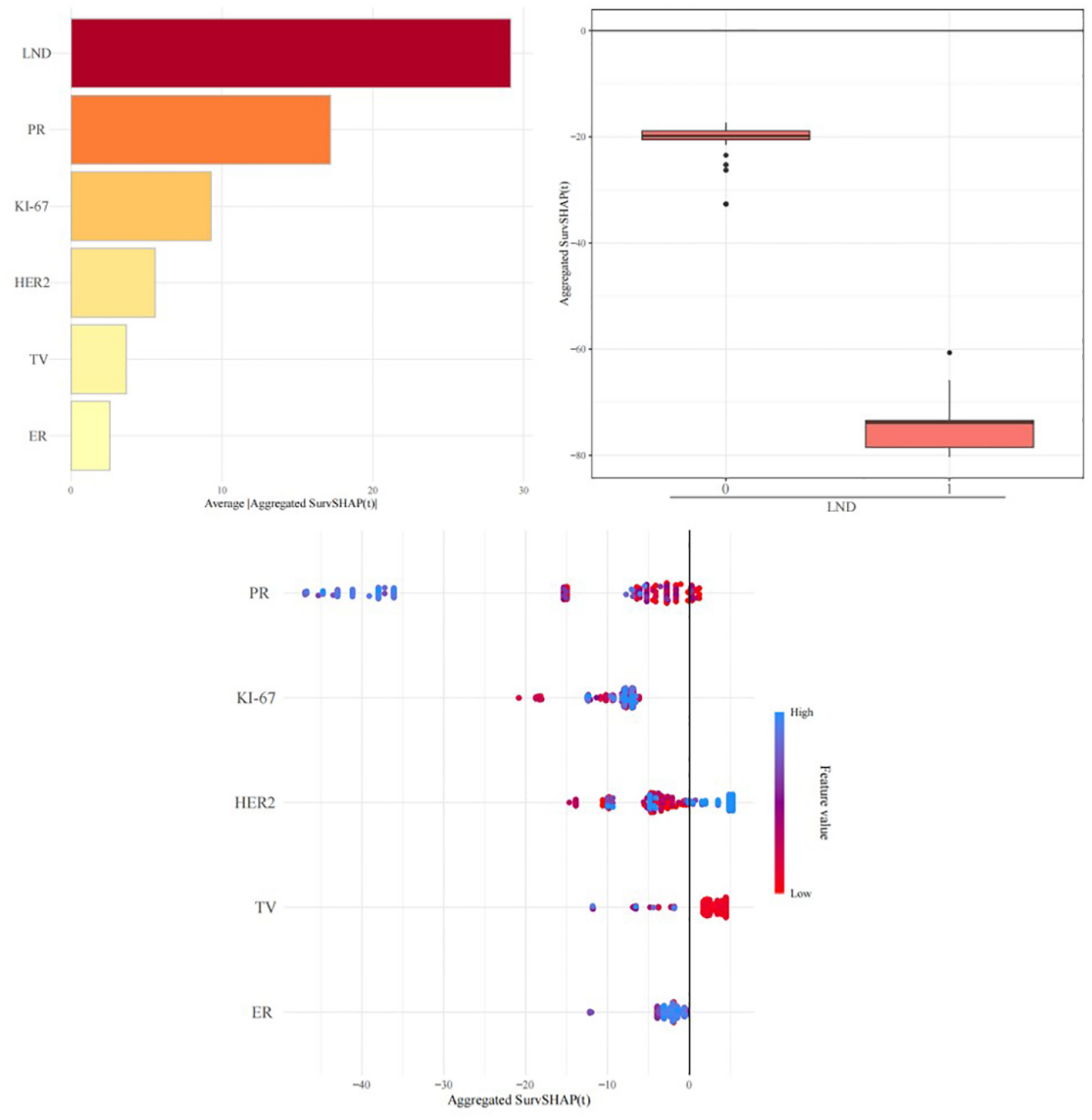
SHAP value distribution (decision tree model).

Significant survival differences between risk groups were observed (p<0.0001) (**Figure 26**). The Decision Tree model achieved training set time-dependent AUCs of 0.736 (1-year), 0.705 (3-year), and 0.827 (5-year) (**Figure 27**). Validation set performance was comparable with AUCs of 0.753 (1-year), 0.750 (3-year), and 0.822 (5-year), showing improved performance with longer follow-up. Calibration curves (**Figure 28**), decision curve analysis (**Figure 29**), and SHAP value distribution (**Figure 30**) are shown.

### Random forest model development and evaluation

**Figure 31:**
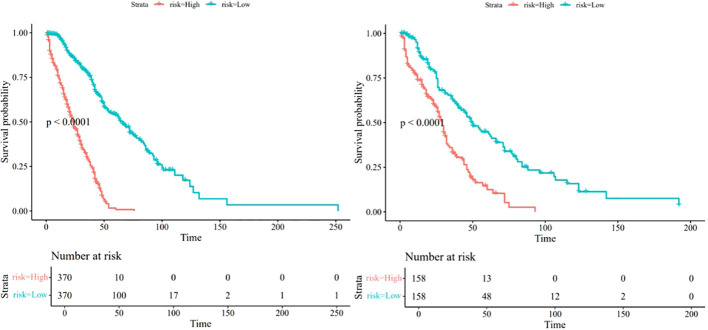
Kaplan-Meier survival curves (random forest model).

**Figure 32:**
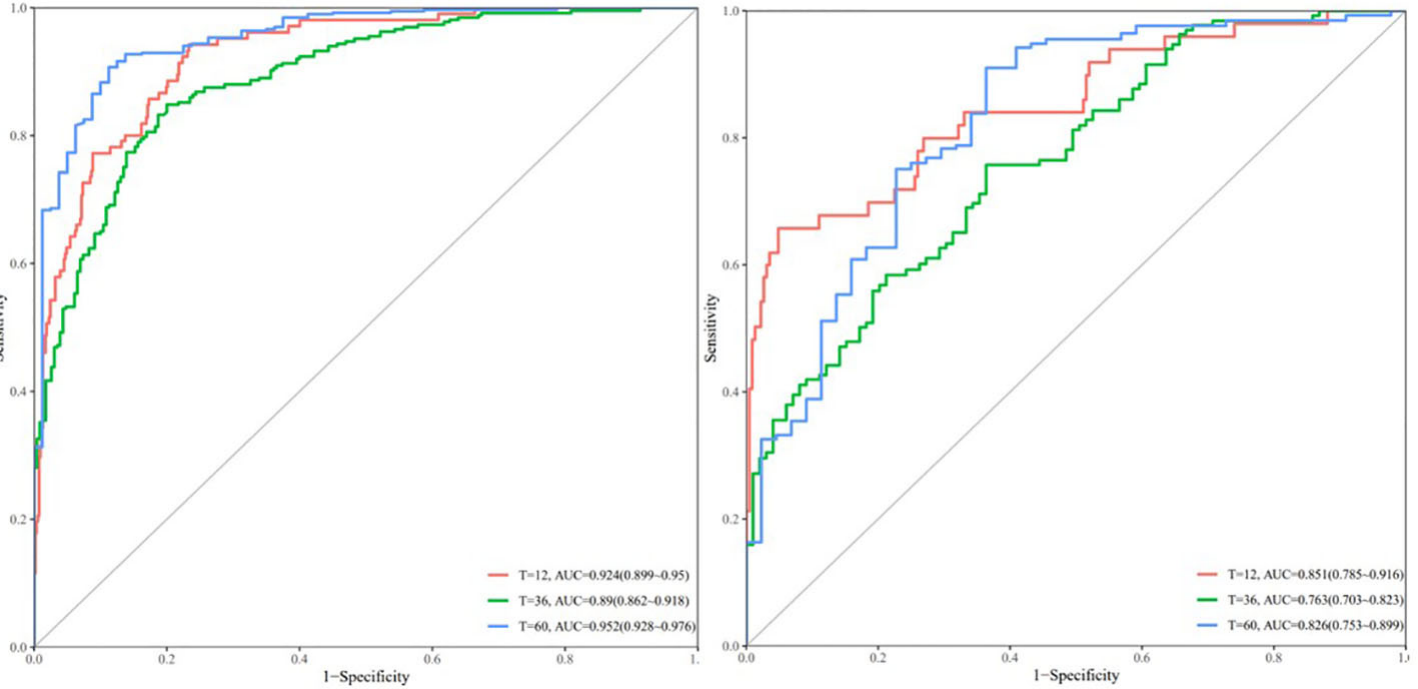
Time-dependent ROC curves (random forest model).

**Figure 33:**
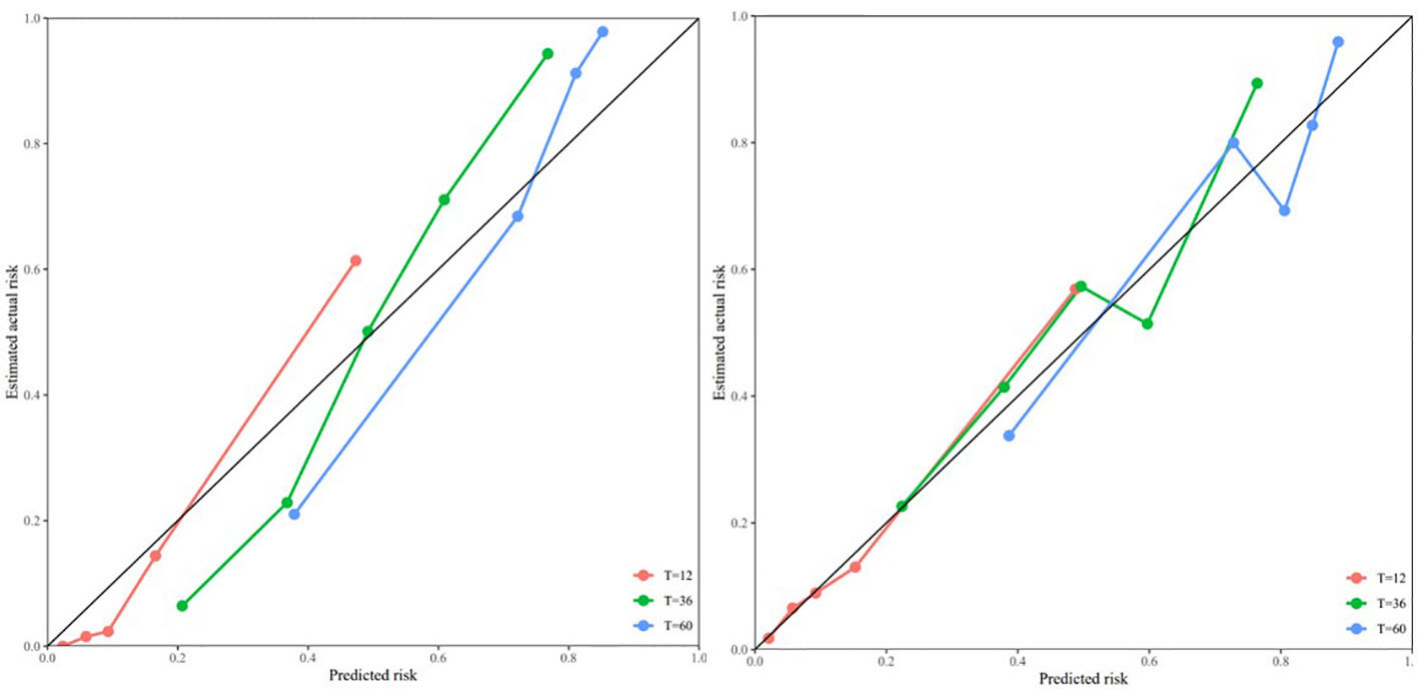
Calibration curves (random forest model).

**Figure 34:**
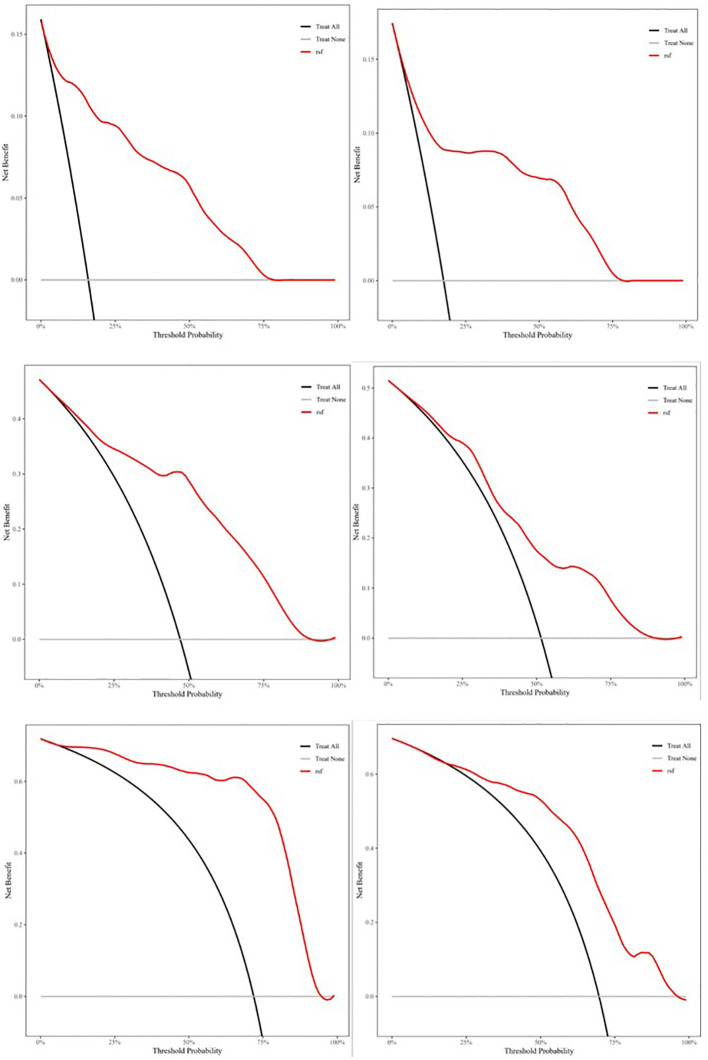
Decision curve analysis (random forest model).

**Figure 35:**
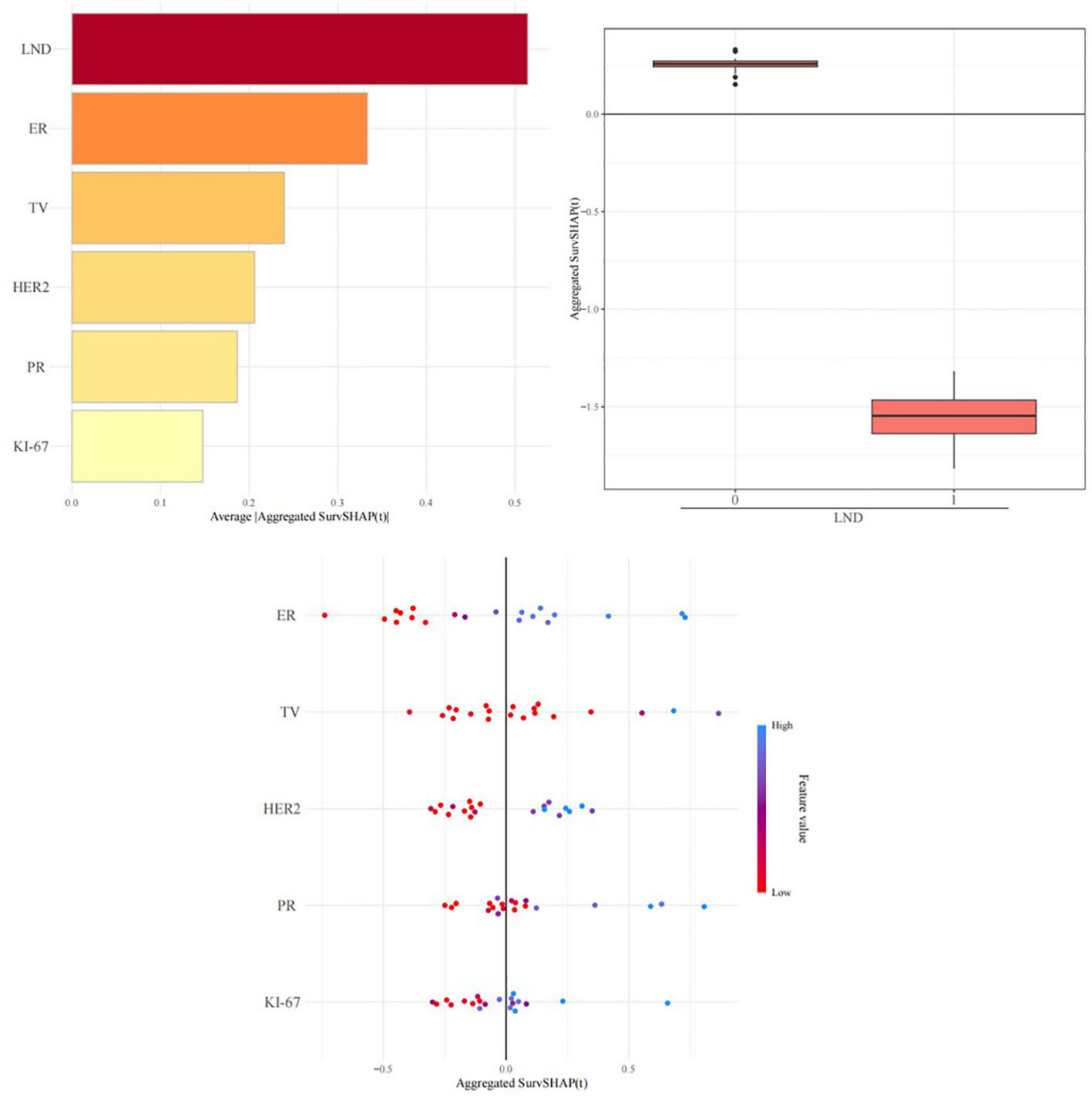
SHAP value distribution (random forest model).

Kaplan-Meier analysis confirmed significant risk stratification (p<0.0001) (**Figure 31**). The Random Forest model demonstrated superior discrimination with training set time-dependent AUCs of 0.924 (1-year), 0.890 (3-year), and 0.952 (5-year) (**Figure 32**). Validation set performance remained strong with AUCs of 0.851 (1-year), 0.763 (3-year), and 0.826 (5-year), confirming robust generalization. The C-index of 0.803 (95%CI: 0.762-0.844) in the validation set further supported its superior overall discriminative capacity. Calibration curves (**Figure 33**), decision curve analysis (**Figure 34**), and SHAP value distribution (**Figure 35**) are presented.

## Discussion

This single-center, retrospective study systematically developed and compared multiple machine learning models for breast cancer prognosis. Our results identified tumor size, ER, PR, Ki-67, and HER2 status as important prognostic factors, consistent with previous studies emphasizing the role of clinicopathological features (24, 25). Importantly, ensemble learning methods, particularly Random Forest, demonstrated superior and stable predictive power. The central methodological contribution of this work is the establishment of a rigorous analytical pipeline—from time-to-event-based feature selection to cross-validated model training and interpretability analysis—which provides a reproducible template for future research in primary healthcare settings. However, all findings must be interpreted with caution given the study’s limitations.

Tumor size is a classic prognostic factor in breast cancer, widely used for risk assessment (26). Numerous studies have demonstrated associations between tumor size and survival, recurrence, and metastasis risk (27). Specifically, larger tumors are typically associated with higher lymph node metastasis rates and poorer survival outcomes (28). The finding that smaller tumor size by ultrasound was associated with poorer outcomes in our study is counterintuitive but has been observed in specific contexts. A plausible explanation is confounding by indication and differences in assessment modality. In our cohort, patients with smaller tumors on ultrasound often had aggressive biology (e.g., high-grade, HER2-positive, or triple-negative subtypes) that might not manifest as large masses, leading to early metastasis. Additionally, smaller palpable tumors might be more amenable to breast-conserving surgery, where pathological tumor size measurement differs from whole-specimen assessment in mastectomy, potentially introducing systematic measurement bias. This highlights the complex relationship between tumor dimension and biological aggressiveness.

In this study, ER and HER2 status were identified as core prognostic factors affecting DFS. Consistent evidence shows that ER-positive patients generally have better outcomes due to responsiveness to endocrine therapy (29, 30). HER2 positivity, historically an aggressive phenotype, remained a key prognostic factor; however, its predictive weight (17.6% SHAP contribution) also reflects the profound impact of modern targeted therapy. Our model captured the independent prognostic value of HER2-low expression, a finding of contemporary interest potentially linked to the heterogeneous efficacy of novel antibody-drug conjugates (31). Future studies with detailed treatment data are needed to dissect the interplay between target expression, therapy received, and survival.

Ki-67’s high contribution (22.1%) to model predictions underscores its core function in cell cycle regulation. Previous studies have primarily focused on linear associations between Ki-67 and proliferation index, with research showing that Ki-67>30% increases breast cancer recurrence risk (32). Through SHAP interaction analysis, we could quantify its non-linear relationship with molecular subtypes, finding that the effect of high Ki-67 was more prominent in Luminal B patients compared to those with triple-negative disease. This subtype-dependent effect may stem from the specific efficacy of CDK4/6 inhibitors in Luminal tumors or a ceiling effect of a high basal proliferation rate in triple-negative disease (33, 34).

The study also highlighted the role of the inflammatory microenvironment. While NLR, PLR, and SII were investigated, their contribution to the final models was limited, and only SII showed considerable weight (12.3%). This selective, rather than universal, contribution suggests that systemic inflammatory markers may reflect a specific aspect of tumor biology, such as M2 macrophage polarization in Luminal subtypes, rather than being a general driver of poor prognosis (35). The limited integration of the full set of inflammatory markers into our final models is an important finding that must be acknowledged, and their practical utility over traditional variables warrants further study in richer datasets.

Furthermore, we acknowledge the inherent limitation of selecting predictors based on multivariable Cox P-values. Such an approach can be sensitive to sample size and may omit borderline variables that carry non-linear or interactive predictive information. Although our supplementary LASSO analysis largely converged on the same set of features, future studies would benefit from employing stability selection or fully embedded feature selection within the machine learning framework to enhance robustness.

### Observational studies are inherently limited in establishing causality

We want to clearly state that terms like “association” or “correlation” are used throughout this manuscript intentionally to avoid any implication of a causal pathway. The observed lower risk associated with lymph node dissection, for example, should not be misinterpreted as a “protective effect” of the procedure, as this association is heavily confounded by clinical decision-making, tumor stage, adjuvant therapy, and other factors that influence both the decision to perform dissection and patient outcomes (36).

### The limitations of this study must be placed at the forefront of interpretation

The single-center, retrospective design is a major constraint on generalizability. The absence of an external validation cohort prevents assessment of model performance in different clinical settings and ethnic groups. Furthermore, potential residual confounding from unmeasured variables, such as detailed chemotherapy regimens, comorbidities, or nuanced staging parameters, cannot be ruled out despite the use of multivariable adjustment. Higher prediction error rates in young patients (≤30 years) suggest the need for age-specific models (37). Therefore, all claims regarding clinical applicability must be tempered. Our models represent “promising tools under development,” not a “reliable tool for clinical decision-making.” The statement about “redefining clinical standards” has been removed as it is not supported by the current level of evidence. Future research will focus on three pillars of model advancement: (1) rigorous external validation through multi-center cohorts; (2) integration of higher-dimensional data like spatial transcriptomics or advanced imaging; and (3) development of dynamic prognosis systems based on ctDNA mutation abundance for real-time monitoring of treatment response (38, 39). Until such validation is complete, our work should be viewed as a methodological step forward, not a clinical solution.

## Conclusion

This single-center study identifies key prognostic factors for breast cancer and demonstrates that ensemble machine learning models, particularly Random Forest, offer superior predictive performance for short- and long-term outcomes when evaluated with a time-dependent framework and rigorous internal validation. SHAP-based interpretation enhances model transparency. However, given the single-center retrospective design, lack of external validation, and potential for residual confounding, the developed models cannot yet be considered a reliable or generalizable clinical tool. Future multi-center, prospective studies with independent external validation are essential to confirm these findings before any clinical translation can be considered. This work provides a certain methodological foundation for such future investigations.

## Data Availability

The original contributions presented in the study are included in the article/supplementary material. Further inquiries can be directed to the corresponding author.

## References

[1] Sung H Ferlay J Siegel RL Laversanne M Soerjomataram I Jemal A . Global cancer statistics 2020: GLOBOCAN estimates of incidence and mortality worldwide for 36 cancers in 185 countries. CA Cancer J Clin. (2021) 71:209–49. doi: 10.3322/caac.21660 33538338

[2] Bray F Ferlay J Soerjomataram I Siegel RL Torre LA Jemal A . Global cancer statistics 2018: GLOBOCAN estimates of incidence and mortality worldwide for 36 cancers in 185 countries. CA Cancer J Clin. (2018) 68:394–424. doi: 10.3322/caac.21492 30207593

[3] Waks AG Winer EP . Breast cancer treatment: a review. JAMA. (2019) 321:288–300. doi: 10.1001/jama.2018.19323 30667505

[4] Harbeck N Gnant M . Breast cancer. Lancet. (2017) 389:1134–50. doi: 10.1038/nrclinonc.2011.214 27865536

[5] Goldhirsch A Winer EP Coates AS Gelber RD Piccart-Gebhart M Thürlimann B . Personalizing the treatment of women with early breast cancer: highlights of the St Gallen International Expert Consensus on the Primary Therapy of Early Breast Cancer 2013. Ann Oncol. (2013) 24:2206–23. doi: 10.1093/annonc/mdt303 23917950 PMC3755334

[6] Cianfrocca M Goldstein LJ . Prognostic and predictive factors in early-stage breast cancer. Oncologist. (2004) 9:606–16. doi: 10.1634/theoncologist.9-6-606 15561805

[7] Rakha EA Reis-Filho JS Baehner F Dabbs DJ Decker T Eusebi V . Breast cancer prognostic classification in the molecular era: the role of histological grade. Breast Cancer Res. (2010) 12:207. doi: 10.1186/bcr2607 20804570 PMC2949637

[8] Phung MT Tin Tin S Elwood JM . Prognostic models for breast cancer: a systematic review. BMC Cancer. (2019) 19:230. doi: 10.1186/s12885-019-5442-6 30871490 PMC6419427

[9] Hanahan D Weinberg RA . Hallmarks of cancer: the next generation. Cell. (2011) 144:646–74. doi: 10.1016/j.cell.2011.02.013 21376230

[10] Mantovani A Allavena P Sica A Balkwill F . Cancer-related inflammation. Nature. (2008) 454:436–44. doi: 10.1016/b978-0-12-801238-3.65801-4 18650914

[11] Templeton AJ McNamara MG Šeruga B Vera-Badillo FE Aneja P Ocaña A . Prognostic role of neutrophil-to-lymphocyte ratio in solid tumors: a systematic review and meta-analysis. J Natl Cancer Inst. (2014) 106:dju124. doi: 10.1093/jnci/dju124 24875653

[12] Ethier JL Desautels D Templeton A Shah PS Amir E . Prognostic role of neutrophil-to-lymphocyte ratio in breast cancer: a systematic review and meta-analysis. Breast Cancer Res. (2017) 19:2. doi: 10.1186/s13058-016-0794-1 28057046 PMC5217326

[13] Grivennikov SI Greten FR Karin M . Immunity, inflammation, and cancer. Cell. (2010) 140:883–99. doi: 10.1016/j.cell.2010.01.025 20303878 PMC2866629

[14] Obermeyer Z Emanuel EJ . Predicting the future - big data, machine learning, and clinical medicine. N Engl J Med. (2016) 375:1216–9. doi: 10.1056/nejmp1606181 27682033 PMC5070532

[15] Deo RC . Machine learning in medicine. Circulation. (2015) 132:1920–30. doi: 10.1161/circulationaha.120.050583 26572668 PMC5831252

[16] Sidey-Gibbons JAM Sidey-Gibbons CJ . Machine learning in medicine: a practical introduction. BMC Med Res Methodol. (2019) 19:64. doi: 10.1186/s12874-019-0681-4 30890124 PMC6425557

[17] Rajkomar A Dean J Kohane I . Machine learning in medicine. N Engl J Med. (2019) 380:1347–58. doi: 10.1056/nejmra1814259 30943338

[18] Kourou K Exarchos TP Exarchos KP Karamouzis MV Fotiadis DI . Machine learning applications in cancer prognosis and prediction. Comput Struct Biotechnol J. (2015) 13:8–17. doi: 10.1016/j.csbj.2014.11.005 25750696 PMC4348437

[19] Cruz JA Wishart DS . Applications of machine learning in cancer prediction and prognosis. Cancer Inform. (2007) 2:59–77. doi: 10.1177/117693510600200030 19458758 PMC2675494

[20] Esteva A Kuprel B Novoa RA Ko J Swetter SM Blau HM . Dermatologist-level classification of skin cancer with deep neural networks. Nature. (2017) 542:115–8. doi: 10.1038/nature21056 28117445 PMC8382232

[21] Hosny A Parmar C Quackenbush J Schwartz and Aerts LHHJ . Artificial intelligence in radiology. Nat Rev Cancer. (2018) 18:500–10. doi: 10.1038/s41568-018-0016-5 29777175 PMC6268174

[22] Lundberg SM Lee SI . A unified approach to interpreting model predictions. Adv Neural Inf Process Syst. (2017) 30:4765–74. doi: 10.48550/arXiv.1705.07874

[23] Collins GS Reitsma JB Altman DG Moons KG . Transparent reporting of a multivariable prediction model for individual prognosis or diagnosis (TRIPOD): the TRIPOD statement. BMJ. (2015) 350:g7594. doi: 10.1016/j.jclinepi.2014.11.010 25569120

[24] Yu X Zhang Z Wang Z Wu P Qiu and Huang FJ . Prognostic value of tumor size in breast cancer: a systematic review. Breast Cancer Res Treat. (2020) 179:287–98. doi: 10.1007/s12094-015-1391-y 26459255 PMC4823351

[25] Liu J Guo H Mao K Zhang K Deng H Liu Q . Estrogen receptor status and prognosis in breast cancer: a meta-analysis. J Clin Oncol. (2018) 36:4520–9. doi: 10.1007/s10549-016-3721-3

[26] Hayes DF Isaacs C Stearns V . Prognostic factors in breast cancer: current and new predictors of metastasis. J Mammary Gland Biol Neoplasia. (2001) 6:375–92. doi: 10.1023/a:1014778713034 12013528

[27] Carter CL Allen C Henson DE . Relation of tumor size, lymph node status, and survival in 24,740 breast cancer cases. Cancer. (1989) 63:181–92. doi: 10.1002/1097-0142(19890101)63:1<181::aid-cncr2820630129>3.0.co;2-h 2910416

[28] Narod SA . Tumour size predicts long-term survival among women with lymph node-positive breast cancer. Curr Oncol. (2012) 19:249–53. doi: 10.3747/co.19.1043 23144572 PMC3457875

[29] Early Breast Cancer Trialists' Collaborative Group (EBCTCG) . Effects of chemotherapy and hormonal therapy for early breast cancer on recurrence and 15-year survival: an overview of the randomised trials. Lancet. (2005) 365:1687–717. doi: 10.1016/S0140-6736(05)66544-0 15894097

[30] Davies C Godwin J Gray R Clarke M Cutter D Darby S . Relevance of breast cancer hormone receptors and other factors to the efficacy of adjuvant tamoxifen: patient-level meta-analysis of randomised trials. Lancet. (2011) 378:771–84. doi: 10.1016/S0140-6736(11)60993-8 PMC3163848 21802721

[31] Modi S Jacot W Yamashita T Sohn J Vidal M Tokunaga E . Trastuzumab deruxtecan in previously treated HER2-low advanced breast cancer. N Engl J Med. (2022) 387:9–20. doi: 10.1056/nejmoa2203690 35665782 PMC10561652

[32] Petrelli F Viale G Cabiddu M Barnil S . Prognostic value of Ki-67 in breast cancer: a systematic review and meta-analysis. Breast Cancer Res Treat. (2015) 152:487–98. doi: 10.1007/s10549-015-3559-0 26341751

[33] Finn RS Martin M Rugo HS Jones S Im SA Gelmon K . Palbociclib and letrozole in advanced breast cancer. N Engl J Med. (2016) 375:1925–36. doi: 10.1056/nejmoa1607303 27959613

[34] Hortobagyi GN Stemmer SM Burris HA Yap YS Sonke GS Paluch-Shimon S . Ribociclib as first-line therapy for HR-positive, advanced breast cancer. N Engl J Med. (2016) 375:1738–49. doi: 10.1056/nejmoa1609709 27717303

[35] Proctor MJ Morrison DS Talwar D Balmer SM Fletcher CD O’Reilly D . A comparison of inflammation-based prognostic scores in patients with cancer. A Glasgow Inflammation Outcome Study. Eur J Cancer. (2011) 47:2633–41. doi: 10.1016/j.ejca.2011.03.028 21724383

[36] Giuliano AE Ballman KV Mccall L Beitsch PD Brennan MB Kelemen PR . Effect of axillary dissection vs no axillary dissection on 10-year overall survival among women with invasive breast cancer and sentinel node metastasis: the ACOSOG Z0011 (Alliance) randomized clinical trial. JAMA. (2017) 318:918–26. doi: 10.1001/jama.2017.11470 28898379 PMC5672806

[37] Fredholm H Eaker S Frisell J Holmberg L Fredriksson and Lindman IH . Breast cancer in young women: poor survival despite intensive treatment. PloS One. (2009) 4:e7695. doi: 10.1371/journal.pone.0007695 19907646 PMC2770847

[38] Collins GS Ogundimu EO Altman DG . Sample size considerations for the external validation of a multivariable prognostic model: a resampling study. Stat Med. (2016) 35:214–26. doi: 10.1002/sim.6787 26553135 PMC4738418

[39] Heitzer E Haque IS Roberts CES Speicher MR . Current and future perspectives of liquid biopsies in genomics-driven oncology. Nat Rev Genet. (2019) 20:71–88. doi: 10.1038/s41576-018-0071-5 30410101

